# Multilevel strategies to end HIV for young couples in Cape Town: Study protocol for a cluster randomized trial

**DOI:** 10.1371/journal.pone.0305056

**Published:** 2024-06-07

**Authors:** Wendee M. Wechsberg, Tara Carney, Felicia A. Browne, Isa M. van der Drift, Tracy L. Kline, Laura L. Nyblade, Jacqueline Ndirangu, Catherine Orrell, Courtney P. Bonner, Emily Caron

**Affiliations:** 1 RTI International, Research Triangle Park, Durham, NC, United States of America; 2 Gillings School of Global Public Health, University of North Carolina, Chapel Hill, NC, United States of America; 3 Psychiatry and Behavioral Sciences, Duke University School of Medicine, Durham, NC, United States of America; 4 Mental Health, Alcohol, Substance Use and Tobacco Research Unit, South African Medical Research Council, Tygerberg, South Africa; 5 Department of Psychiatry and Mental Health, Division of Addiction Psychiatry, University of Cape Town, Groote Schuur Hospital, Observatory, Cape Town, South Africa; 6 RTI International, Washington, DC, United States of America; 7 RTI International, Nairobi, Kenya; 8 UCT Faculty of Health Sciences, Desmond Tutu Health Foundation, Observatory, Cape Town, South Africa; PLOS: Public Library of Science, UNITED KINGDOM

## Abstract

This protocol presents a multilevel cluster randomized study in 24 communities in Cape Town, South Africa. The study comprises four specific aims. Aim 1, conducted during the formative phase, was to modify the original Couples Health CoOp (CHC) intervention to include antiretroviral therapy/pre-exposure prophylaxis (ART/PrEP), called the Couples Health CoOp Plus (CHC+), with review from our Community Collaborative Board and a Peer Advisory Board. Aim 1 has been completed for staging the trial. Aim 2 is to evaluate the impact of a stigma awareness and education workshop on community members’ attitudes and behaviors toward young women and men who use AODs and people in their community seeking HIV services (testing/ART/PrEP) and other health services in their local clinics. Aim 3 is to test the efficacy of the CHC+ to increase both partners’ PrEP/ART initiation and adherence (at 3 and 6 months) and to reduce alcohol and other drug use, sexual risk and gender-based violence, and to enhance positive gender norms and communication relative to HIV testing services (n = 480 couples). Aim 4 seeks to examine through mixed methods the interaction of the stigma awareness workshop and the CHC+ on increased PrEP and ART initiation, retention, and adherence among young women and their primary partners. Ongoing collaborations with community peer leaders and local outreach staff from these communities are essential for reaching the project’s aims. Additionally, a manualized field protocol with regular training, fidelity checks, and quality assurance are critical components of this multilevel community trial for successful ongoing data collection.

**Trial registration.** Clinicaltrials.gov Registration Number: NCT05310773. Pan African Trials: pactr.samrc.ac.za/ Registration Number: PACTR202205640398485.

## Introduction

South Africa continues to report high levels of alcohol consumption, increasing opiate use, and particularly high rates of methamphetamine and methaqualone use in certain areas [1–4]. Alcohol and other drug (AOD) use undermines women’s personal agency in negotiating condom use with their sexual partners and accessing healthcare services, posing barriers to ending the HIV epidemic [5–7]. The intersection of AOD use, sexual risk behavior, and gender-based violence (GBV) in South Africa creates a complex syndemic that increases HIV risk [7–9]. In South Africa, a historical backdrop of gender inequality within a patriarchal society has compounded women’s lack of sexual autonomy [10]. Continued heavy AOD use among young women and their partners exacerbates the risk of HIV transmission by contributing to outside sexual partnerships, GBV, impaired condom use, and reduced adherence to antiretroviral therapy (ART) [11]. HIV treatment as prevention, particularly ART and pre-exposure prophylaxis (PrEP) for protecting partners against HIV, has shown promising results in reducing HIV transmission [12]. However, successful implementation of ART and PrEP requires addressing relational and contextual factors and structural challenges [11].

The highest HIV incidence among women in South Africa occurs during young adulthood, often in the context of primary partnerships characterized by AOD use, condomless sex, and outside sexual partnerships [8,13]. Consequently, engaging male partners in the HIV continuum is crucial to support HIV prevention efforts [14,15]. While previous research has focused on age-disparate or intergenerational sexual relationships, there is a need to address the health and behaviors of primary male partners in tandem with young women [16,17].

Couples-based interventions have demonstrated efficacy in improving ART adherence and show promise in enhancing PrEP adherence [18,19]. By involving both partners, these interventions can effectively reduce HIV transmission [14,20,21]. For example, the original Couples Health CoOp (CHC), an empowerment-based intervention developed for young couples in South African, addressed AOD use, GBV, and sexual risk within relationships [22]. The CHC has shown positive outcomes for condom use, gender norms, and reducing HIV incidence among women [18,22]. Long-term outcomes included improved communication between couples [23]. However, the original CHC did not specifically address ART or PrEP uptake and adherence, highlighting the need for this modification.

Structural challenges also exist where young couples live. Stigma within communities has also been identified as a barrier to health services among people who use AODs or are living with HIV and for young women who are sexually active [24–26]. Adaptations of stigma and discrimination training directed by community members has been found to be effective [27–29].

This article presents the protocol for a cluster randomized trial aimed at evaluating the Couples Health CoOp Plus (CHC+) as a newly enhanced intervention that integrates ART/PrEP initiation and adherence with the existing CHC model and a structural intervention of stigma awareness workshops in communities. CHC+ incorporates added information on sexual and reproductive health, a status-neutral approach, treatment as prevention (U = U), and the significance of ART and PrEP.

To address structural factors, the study team developed a stigma reduction curriculum based on a stigma reduction toolkit and a community HIV prevention project [30] with community peers for a large community awareness with Stop and Go skits to tackle stigmatizing attitudes and behaviors toward young women and men who use AODs and who may need health services, including HIV treatment and prevention.

The research adopts a multilevel social ecological framework, emphasizing individual, couple, and structural factors in HIV risk and access to services [31,32]. The overarching goal is to implement a more comprehensive approach that addresses the intersecting epidemics of AOD use, sexual risk behavior, and HIV transmission among young couples in South Africa. By including ART/PrEP initiation and adherence, a stigma awareness workshop, in a multilevel approach, the study aims to enhance HIV prevention efforts, improve treatment outcomes, and alleviate the burden of HIV within this key population. The study findings hold the potential to guide future interventions and contribute significantly to helping end the HIV epidemic in South Africa.

### Study aims

**Fig 1:**
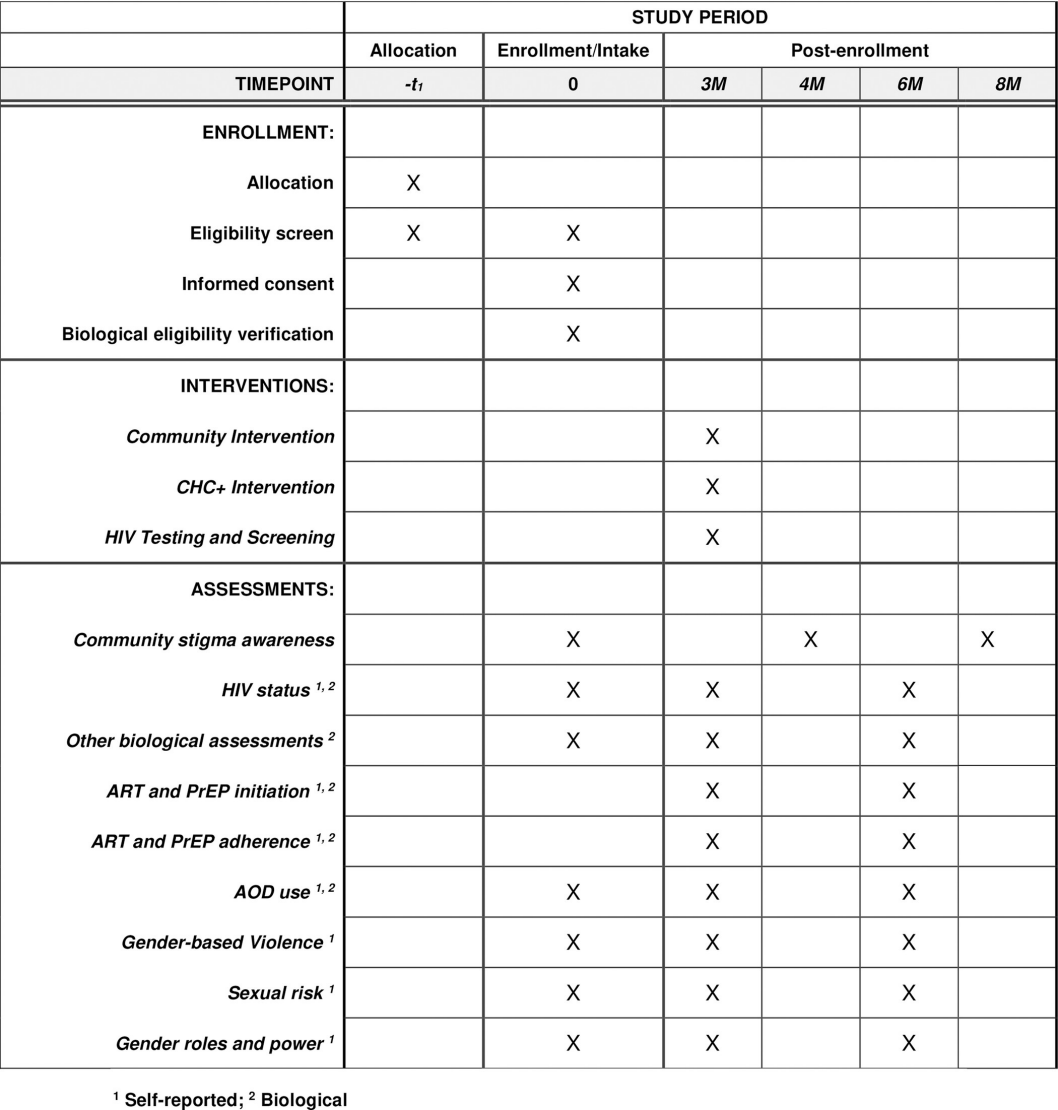
Schedule of enrollment, interventions, and assessments.

This study comprises four specific aims. Aim 1, conducted during the formative phase, was to modify the original CHC intervention to include ART/PrEP with review from our Community Collaborative Board (CCB) and a Peer Advisory Board (PAB). Aim 2 is to evaluate the impact of a stigma awareness and education workshop on community members’ attitudes and behaviors toward young women and men who use AODs and other people seeking HIV services (testing/ART/PrEP) and other health services. Aim 3 is to test the efficacy of the CHC+ to increase both partners’ PrEP/ART initiation and adherence (at 3 and 6 months) and reduce AOD use, sexual risk and GBV, and enhance positive gender norms and communication relative to HTS. Lastly, Aim 4 seeks to examine through mixed methods the interaction of the stigma awareness workshop and the CHC+ on increased PrEP and ART initiation, retention, and adherence among young women and their primary partners (see **Fig 1** for SPIRIT schedule).

#### Conceptual framework

**Fig 2:**
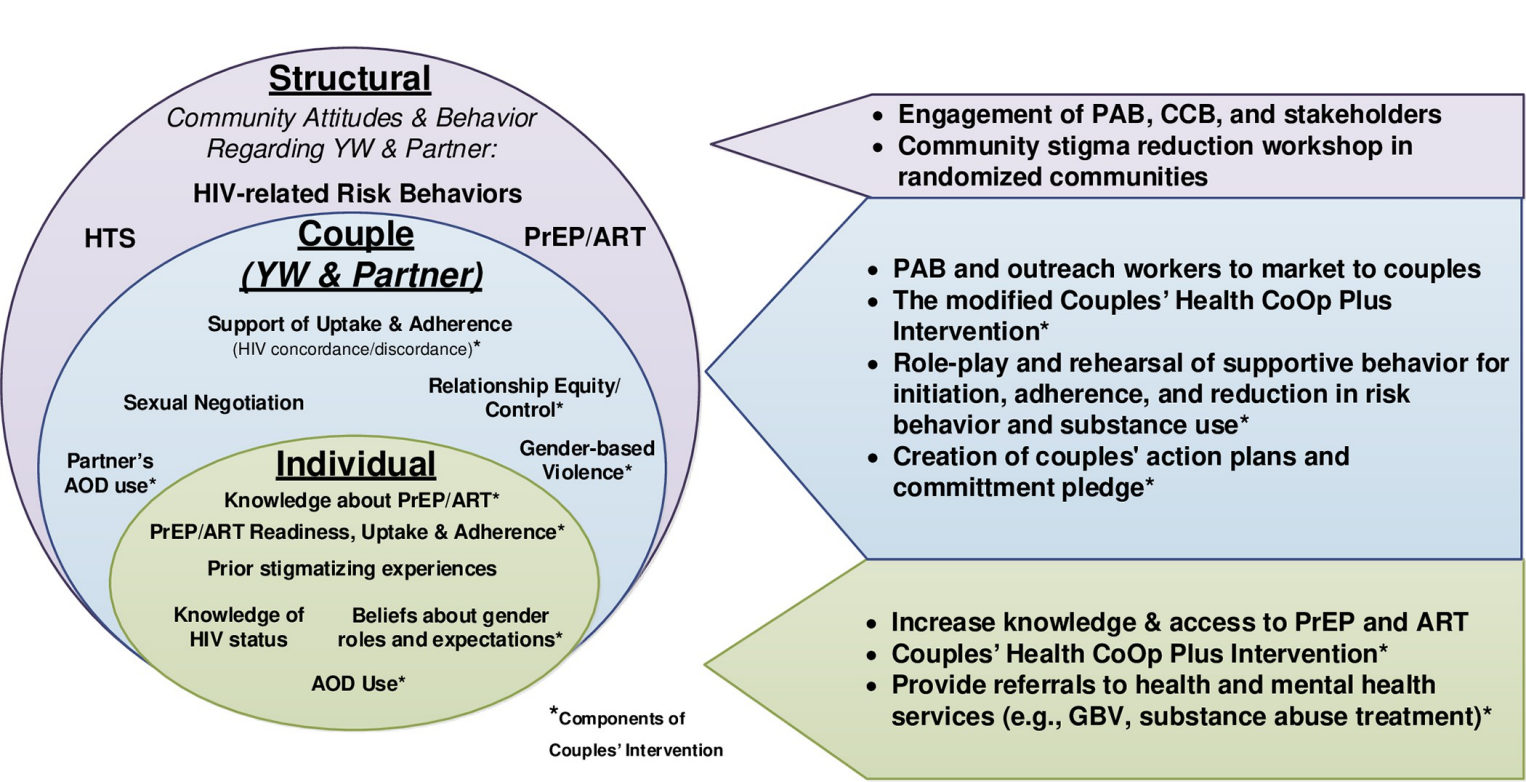
Social ecological framework and strategies.

**Fig 2** presents a comprehensive conceptual framework reflecting the multiple levels of the social ecological framework and the strategies to accomplish the aims for the individual, as a couple, and structural levels within their environment. The framework also incorporates the supports and challenges needed to address the multiple processes that will be used to accomplish the study aims.

## Materials and methods

The protocol for this study has been approved by the following health research ethics committees: South African Medical Research Council (primary institution: EC031-8/2020) and RTI International (defers to primary institution for approval).

### Setting and study design

#### Setting

City Health Clinics within or adjacent to historically disadvantaged communities in the Cape Town area that were selected for the government PrEP rollout were identified. After mapping these clinics, the clinics that were not accessible to potential participants due to distance were removed, resulting in a total of 24 clinic catchment areas or communities. The communities were classified by population groups, including Black, Coloured (of mixed ancestry—an official term used in South Africa for population classification), and some communities with both population groups.

#### Study research design

**Fig 3:**
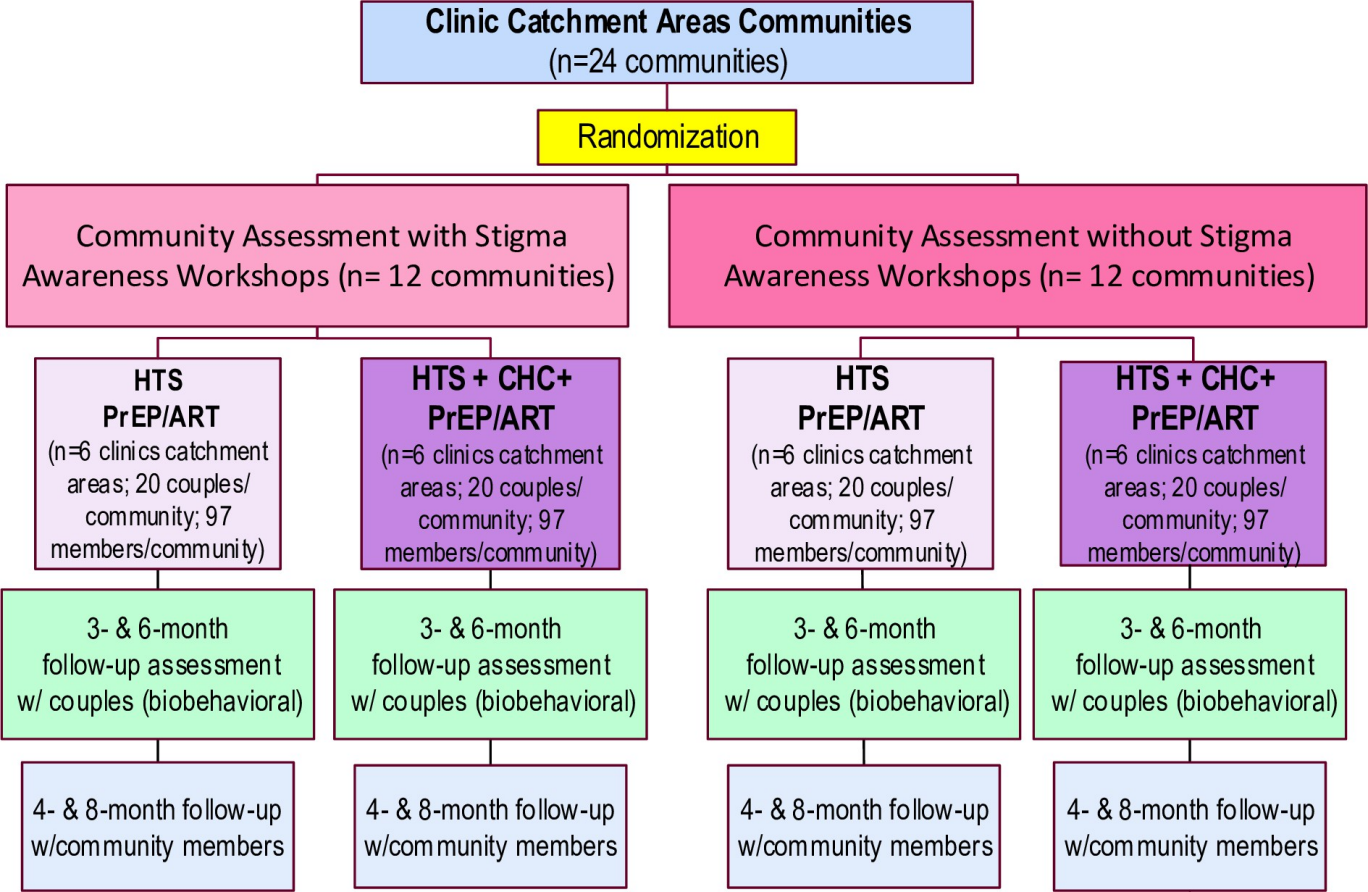
Multilevel research design.

This study is a four-arm cluster randomized trial that has two interventions: a community stigma awareness workshop (for communities) and the CHC+ (for couples). Specifically, 24 Cape Town communities located near clinics that provide ART and PrEP were randomized into one of four arms (see **Fig 3**): (1) stigma awareness workshop (community) and HTS/PrEP/ART (couples); (2) stigma awareness workshop (community), and HTS/PrEP/ART with the CHC+ (couples); (3) no stigma awareness workshop (community) and HTS/PrEP/ART (couples; control); and (4) no stigma awareness workshop (community), and HTS/PrEP/ART with the CHC+ (couples). Twenty couples (a total of 480 couples), defined as young women and their primary male partners both aged 18 to 30 are recruited from each community. Depending on the study arm of their community, couples are provided with HTS/PrEP/ART and/or the CHC+. Couples are asked to return for two study follow-up appointments at 3- and 6-months post-enrollment. Communities are already assigned to receive either the stigma awareness workshop or no workshop, with baseline and repeated follow-up surveys with community members. Recruitment began in July 5, 2022 and is ongoing.

#### Randomization

To achieve balance in allocation of population groups across the four study arms, stratified randomization by population group (Black African, Coloured, and Mixed) was conducted. A total of 24 catchment areas were selected from the list of 34 catchment areas. The sampling frame included 8 Black African, 8 Coloured and 8 Mixed communities. Within each of the 3 strata, the 8 areas were randomized to the four study arms in a 2:2:2:2 allocation.

#### Sample size

A sample size of 480 couples, 120 per each of the four study arms, was chosen to ensure sufficient statistical power to detect meaningful differences in our primary outcomes of couple-level ART/PrEP initiation and adherence between intervention and control groups. These power calculations assume 80% power, and an intraclass correlation coefficient (ICC) of 0.05 (based on the team’s previous R01 study with couples in Cape Town) [22], with an estimated follow-up of 95% at 3-month follow-up and 90% at 6-month follow-up. On average, a sample size of 97 community members in each of the 24 catchment areas was calculated to detect a 10% difference between control and intervention conditions on measures of stigma reduction. Using a two-sided chi-square test of proportions with a significance level of 0.05 and nested design model with additivity on the primary intervention effect (stigma awareness community workshop as compared with no community workshop) and secondary intervention effect (CHC+ as compared with HTS), our sample size was selected to ensure power for contrasts based on the presence of an interaction between the community-based stigma awareness workshop and the CHC+. For a hypothesis related to ART/PrEP initiation among couples in the CHC+ arm as compared with couples in the HTS arm, we will be able to detect a minimal effect size of 0.23. The minimal effect size of the CHC+ intervention on ART/PrEP adherence by couples in the CHC+ arm at 6 months is estimated to be 0.34 compared with couples in clinics in the HTS arm. For the evaluation of the efficacy of the stigma awareness workshop plus the CHC+ as compared with no stigma awareness workshop plus HTS (control), the minimal effect size related to ART/PrEP initiation is estimated to be 0.45. For examination of the intervention components’ effects (stigma awareness workshop and the CHC+) on ART/PrEP adherence), the minimal effect size is estimated to be between 0.26 and 0.6 for retention at 3-month and 6-month follow-up.

### Community

#### Community brief survey and workshop recruitment

Prior to conducting any brief community-level survey and workshop activities, trained outreach staff ensure that the study has been adequately introduced to key community leaders or gatekeepers, and that the necessary permission is granted to conduct these activities. During community mapping, they assist with identifying where to conduct assessments and the workshop, and addressing any security issues in the community. Trained outreach staff then identify areas where people congregate in the catchment area, including community malls, centres or halls, street corners, hair and beauty establishments where community members can be recruited for the brief community survey and workshop by distributing marketing flyers and describing the workshop objectives to community members.

#### Eligibility: Brief community survey

To be eligible to complete community surveys, individuals need to be 18 or older, live in the community selected and provide written consent to take part in the survey. Attempts will be made to ensure that the sample is representative of the community. While there is no eligibility criterion around gender, efforts will be made to attain a gender balance. Contact information will not be obtained from participants, as the survey is meant to be anonymous. There is no age restriction to attend the community workshops.

#### Screening: Brief community survey

Staff members will introduce themselves and the study to community members and explain the community assessment. Following this, written informed consent is obtained.

#### Informed consent: Brief community survey

Informed consentcan be completed in a group setting if everyone is comfortable with this, however, if participants are comfortable completing consent in different languages (English, Afrikaans, isiXhosa), then one-on-one consenting may be necessary. If group consenting takes place, each individual needs to understand what they are consenting to and sign an individual consent. Multilingual staff members will assist in this process.

#### Data collection: Community

Baseline data on community stigma are obtained through the administration of a brief self-administered, paper-and-pencil questionnaire to individuals 18 or older residing in all 24 study communities surrounding the study clinics. The sample for the questionnaire is selected through convenience sampling. This questionnaire is a collection of validated stigma measures that have been modified for this study [33,34]. Individuals provide written consent prior to participation in the survey. We will conduct repeated surveys in all 24 communities 4 and 8 months later, through a repeated cross-sectional design to assess stigma awareness from the workshop (described below) and dissemination of information to community members who did not attend the workshop.

#### Stigma awareness workshop

We utilized the Understanding and Challenging HIV Stigma: Toolkit for Action curriculum which focuses on three key actionable drivers of HIV-related stigma: improving understanding of stigma and discrimination, addressing attitudes about HIV transmission, and addressing attitudes toward individuals living with HIV or engaging in risk behaviors for a large community sample [35–37]. This toolkit is designed to be easily adaptable to different contexts. For this study, the curriculum was adapted and simplified with community peer advisory board (PAB) members developing skits for Stop and Go dramas. These skits raise awareness and sensitivity to specifically address and build awareness in the beliefs, attitudes, and behaviors of community members toward young people who use substances and are at risk for HIV acquisition within their communities and address issues that young people face, such as GBV, AOD use, family dynamics, and poor community support. The additional goal is to support treatment (including as HIV prevention) and other healthcare for young couples. The adapted curriculum targets the unique challenges faced by this specific population living in these historically disadvantaged communities and was tailored to their needs and circumstances by PAB members. **Table 1** presents an overview of the community stigma awareness workshop.

**Table 1:** Overview of community workshop on stigma awareness topics.

Activity 1: Welcome and Introduction	Activity 2: Skit on HIV, ART, PrEP, and U = U	Activity 3: Drama	Activity 4: Collective call to action
Energizer: Song or clapping activity to engage participants and help them relax	Skit set in tavern during football match with two young males and young female that discusses: • HIV transmission and protection • Non-stigmatizing terminology around HIV • Myths on HIV transmission • Information on HIV medication (ART) and adherence • Explanation of U = U • Treatment as prevention (PrEP) vs PEP • Medication side-effects • Access to HIV medication at healthcare services • Q&A: HIV and understanding of medication to prevent and treat HIV	Series of topics depicted in skits set in community that includes drivers and effects of stigma that young people may face. Includes a number of characters (peers, boyfriend, parents, neighbours). Addresses the following issues: • Substance use and its effects • Abuse/Gender-based violence • Gender normative roles • Unintended pregnancy • HIV status • Multilevel stigma (family, community)Community poverty • Q&A: on issue depicted in drama, stigma at different levels and effects on young people • Positive outcome: same situation depicted with support and positive attitudes towards young people from family and community • Q&A: different effects on young people and role of community support	• The audience thinks of ways to support young people in their community • Consider how words used can be stigmatizing • Audience members volunteer to share their call to action

#### Implementation of the community stigma awareness workshop

After the brief baseline survey has been completed, community stigma awareness workshops will be conducted in communities that were randomly assigned to receive this stigma awareness activity (N = 12). We expect on average to reach 100 community members to attend these workshops. These workshops can occur within a week of survey completion. The workshops take place in various community settings, such as community library workrooms, churches, sports halls, or temporary tented areas within the community with a minimum of 50 attendees, meaning one or two workshops will be conducted per community. The workshops are facilitated by the field team leads, field staff, and PAB members. Extensive awareness campaigns are conducted within the communities to inform residents about the upcoming workshop, through marketing materials and advertising the workshop with music and a loudspeaker and by word of mouth. These workshops take approximately 2 to 3 hours. Following the workshop, participants can complete a voluntary exit survey that will assess participant satisfaction and elicit suggestions and recommendations on the workshop delivery. The workshop participants are also provided with ART and PrEP information leaflets and resource guides for local services such as substance use rehabilitation centers and mental health services.

### Couples

#### Recruitment: Couples

To generate awareness among young people in the catchment areas surrounding the study clinics, trained outreach staff, comprising male-female pairs, will be strategically deployed. To ensure effective community engagement, the outreach staff will collaborate with the Peer Advisory Board (PAB), a group of young people with deep community connections and relevant lived experience. To minimize bias and protect the privacy and confidentiality of potential participants, PAB members will not be assigned to assist in their own communities. The outreach team will employ various methods, including mapping hotspots and establishing connections with local establishments, shops, salons, and community centers. Skilled and trained outreach staff who reflect the demographics and speak the languages of the study communities are trained in these methods. Recruitment materials, such as recruitment cards and flyers, will be strategically posted within the communities and shared on social media platforms frequently accessed by the target population. By utilizing a combination of in-person and virtual strategies, the outreach team aims to reach a wide range of potential participants and maximize recruitment efforts.

#### Participant eligibility: Couples

To assess eligibility, couples are screened separately but simultaneously in the community. Both individuals are required to meet the following criteria: both partners must be 18 to 30 years old; in a relationship for at least 6 months with the intention of staying together for the next year; report recent condomless sex; report weekly AOD use in the previous 3 months; no PrEP/ART use in the previous 3 months; not currently on medication for multidrug resistant tuberculosis (TB); consent to testing for HIV, and AODs; and for women, not being pregnant at the time of screening as determined by a urine pregnancy test. At least one of the partners also must report having another sexual partner in the past 6 months, with whom they engaged in condomless sex. Limited contact information is collected from eligible couples for scheduling purposes and stored separately from other screening data.

#### Screening: Couples

During the initial contact, individuals will be approached, and verbal consent will be obtained to screen for eligibility. To ensure privacy and confidentiality, the screening process will be conducted individually, with female staff screening the female partner and male staff screening the male partner. In cases where both partners are not present during the initial outreach, screening appointments will be scheduled to facilitate simultaneous screening and determine preliminary eligibility. For the screening process, project staff will use a quick screener form, which allows for efficient and standardized assessment of eligibility criteria. To maintain the integrity of the screening process, staff members will compare answers away from the screened couple to determine initial eligibility. Individuals’ responses are not disclosed to their partner. To respect privacy, couples who are found to be ineligible are not be provided with explanations for their ineligibility, but they will be respectfully informed that they do not meet the criteria for participation. After this initial eligibility screening, biological verification for eligibility is scheduled and conducted at the project site. Both partners are tested for HIV and the female partner is tested for pregnancy. If either partner has an indeterminant HIV test or the female partner has a positive pregnancy test, they are both not eligible.

#### Informed consent: Couples

Eligible couples will be asked to informed consent. To ensure clarity and comprehension, the consent process is conducted separately for each partner within the couple. Study staff members read the consent forms aloud, word for word, to individuals, who request the forms be read in either English, Afrikaans, or isiXhosa. During the consenting process, participants are given the opportunity to ask questions and address any concerns they may have before signing the consent forms. In addition to the general consent forms, a separate release form for accessing relevant medical records is signed, granting permission for staff to review pertinent clinical information. Once individuals consent, they are provided with a card summarizing their rights as study participants and containing contact details for any additional questions or concerns that may arise during their involvement in the study. This promotes ongoing communication and support for the participants throughout their participation in the study. Following informed consent, a locator form is completed to gather accurate participant contact information, and a participant photograph is taken for identification purposes.

#### Data collection: Couples baseline assessments

To conduct the baseline assessments, participants are provided with a questionnaire facilitated by audio computer-assisted self-interviewing (ACASI), available in their preferred language (Afrikaans, English, or isiXhosa). ACASI methodology ensures privacy, minimizes social desirability bias, and enables real-time error checking. Trained staff remain nearby to answer any questions participants may have as they complete the questionnaire. The baseline questionnaire encompasses various key domains, including sexual behavior, substance use, relationship dynamics, experiences of stigma, and access to and utilization of healthcare services. In addition to the questionnaire, participants undergo urine drug screening to assess recent AOD use. This screening aims to provide objective data regarding substance use patterns and support the accuracy of self-reported information. The combined utilization of ACASI and urine drug screening will ensure a robust and reliable data collection process that promotes confidentiality, minimizes response bias, and enhances the overall quality of the baseline assessments.

#### Couples Health CoOp Plus (CHC+) workshops

For couples enrolled in the arms receiving the CHC+ intervention, the workshops are conducted by intervention trained project staff members at convenient community locations, such as community centers, libraries, or other suitable venues. The CHC+ intervention builds on the evidence-based CHC intervention and incorporates a biobehavioral component to promote ART/PrEP initiation and adherence among young women and their main partners [38]. It provides crucial information on PrEP and ART, facilitating linkage to these interventions.

The CHC+ intervention consists of two sessions, with four modules in total, spanning a duration of approximately 2 hours. These sessions aim to enhance knowledge, skills, and agency within the couple, focusing on maintaining overall health, reducing GBV, AOD use, sexual risk behaviors, and ultimately decreasing HIV incidence. The modules cover various topics, including effective communication strategies and risk-reduction methods (see **Table 2**). As supplementary materials, participants receive a handbook containing important information and risk-reduction materials, such as male (external) and female (internal) condoms. Participants in these workshops are also asked to complete a satisfaction form to provide feedback on the intervention.

**Table 2:** Couples Health CoOp Plus (CHC+) workshops.

**Workshop 1**				
***Module 1***		***Module 2***		
*ACTIVITY*: How are men and women treated differently in your community? *ACTIVITY*: Gender equality within couples and HIV prevention. *ACTIVITY*: Pill taking/developing a plan for adherence	• Icebreaker exercises • Importance of reaching young couples and HIV risk • You and your partner as a team together • Positives of being a couple • Gender roles, sexual risks, equality • How do young couples get HIV: facts you need to know • PrEP: A new tool for HIV prevention • ART: How does it work and viral load literacy • U = U • Why is adherence important?	*ACTIVITY*: Uppers and Downer differences *ACTIVITY*: Challenges/solutions to reducing AOD use in couples ACTIVITY: Setting up goals in the Handbook *ACTIVITY*: Practice using male and female condoms *ACTIVITY*: Unwritten rules about condoms	• AOD use, behavior and risk • Facts about AOD use in South Africa • Alcohol levels and risks • Alcohol use and unborn babies • Risk environments • Concerns about dagga, Mandrax • Opiates/injecting and Unga • Uppers such as “Tik” • Reasons to be faithful and challenges • Having a balanced life • Alcohol and drug harm reduction for couples • Benefits of rehab DISCUSS HOMEWORK: Couples sex and relationship activity
**Workshop 2**				
***Module 1***		***Module 2***		
*ACTIVITY*: What is good sex? *ACTIVITY*: Treat vs. Cure (STDs) ACTIVITY: Pleasuring brainstorm activity between couples using speaker/ listener technique	• Risks of STI/STDs • Symptoms and examples of STDs • Staying Healthy • Reducing sexual risks • Male and female anatomy • Condom use: male and female • How to talk with your partner about safer sexy sex • Navigating health services, birth control and family planning • Negotiate about sexy safer sex	*ACTIVITY*: *IDEAL Problem-solving example* *ACTIVITY*: Communicating about family planning ACTIVITY: Harm reduction plans as a couple *ACTIVITY*: Couple Action Plan and Pledge	• Concerns about abuse in South Africa • Violence and sexual assault within couples • Conflict and responses to conflict • Ideal problem-solving and communication • Family planning options and total prevention • Comprehensive response to AOD reduction and HIV prevention for couples • Being positive role models in the community • Circle of safety: monogamy and multiple partners	

#### Data collection: Couples follow-up assessments

Follow-up assessments will be carried out with young couples at 3- and 6-month follow-up intervals to assess participants’ progress and collect relevant data. During these follow-up visits, participants will be requested to reconsent, update their contact information, and complete a follow-up questionnaire utilizing ACASI methodology. This ensures privacy, facilitates accurate responses, and allows for real-time error checking and interpretation assistance, as needed. Biological testing will be conducted to assess HIV status if the partciant was HIV negative at baseline, pregnancy, alcohol use, and other drug use. Participants who report being on and adhering to PrEP or ART will have DBS collected to measure drug concentrations, serving as an objective measure of medication adherence. Participants will be offered a referral to the clinic for ART or PrEP services, ensuring appropriate medical support and treatment. To support participants’ adherence to PrEP or ART regimens and address any concerns or questions that may arise, patient navigation will be provided. This involves regular phone calls and text messages to offer guidance, reinforce medication adherence, and provide ongoing support throughout the study period.

#### Overall study measures

We aim to assess the impact of the stigma awareness workshop on the knowledge, attitudes, and behaviors of the community toward young women and men who are seeking or need health services. Community-level outcomes will be evaluated through repeated measures on their beliefs about people who use alcohol or other drugs, their knowledge about ART and PrEP, their attitudes, beliefs, and behaviors toward people living with HIV, challenges or support from the community to take HIV prevention, and where to go for needed health services and information.

At the couple level, our primary outcomes will include the initiation of ART/PrEP and the documentation of HIV status. We will measure ART/PrEP adherence, as well as PrEP concentration via medical records and dried blood spot (DBS) samples during follow-up appointments. Adherence will also be assessed through self-reported past 30-day use, refills, and the duration of ART or PrEP usage, documented medical records, and DBS. To evaluate substance use, we will conduct baseline and follow-up assessments using rapid urine-based drug screening. This method will detect metabolites of commonly used drugs, providing insight into recent AOD use. Furthermore, we will examine secondary couples-level outcomes, such as self-reports on the frequency of AOD use; experiences of GBV; sexual risk behaviors, including condomless sex; AOD use before sexual encounters; engagement in concurrent partnerships; involvement in sex trade; contraceptive use; and perceptions of HIV risk. We will also assess attitudes toward traditional gender roles, empowerment, assertiveness, and communication between partners regarding testing, status, or sexual relationships. Participants will be asked to self-report experiences of stigma in their communities. To measure these outcomes, we will use a revised risk behavioral assessment (RRBA) [39] that also includes ART/PrEP partner support, treatment readiness, social impact of ART/PrEP use, and health service access and utilization (see **Table 3**).

**Table 3:** Outcome measures for couples and communities.

Outcome	Measures	Time Points Revised
**Aim 2: Stigma awareness and education on community members’ attitudes and behaviors toward young women and men who use AODs and other people seeking HIV services (testing/ART/PrEP) and other health services.**		
Reduction in stigmatizing attitudes and behaviors toward young women and men who use AODs and other people seeking HIV services and other health services.	**Community Stigma Awareness**: Surveys measure stigmatizing attitudes and beliefs toward people who use AODs, people living with HIV and seeking ART, and people seeking PrEP services for prevention. Greater values indicate higher levels of community-level stigma and discrimination.	Baseline, 4 months, and 8 months
Reduced observations of HIV status and AOD use related stigma perpetrated by the community.	**Self-report:** Participant observed or experienced stigma perpetrated by members of their community as measured by the PRBA+.	Baseline, 3 months, and 6 months follow-up (FU)
**Aim 3: Reduce alcohol and other drug use, sexual risk and gender-based violence, and enhance positive gender norms and communication relative to HIV testing services.**		
Reduction in recent drug use (amphetamine, cocaine, methamphetamine, marijuana [THC], opiates and/or Mandrax).	**Biological:** Measured using a rapid urine-based drug screening test panel. **Self-report:** ASSIST frequency of use and RRBA injection use.	Baseline, 3 months, and 6 months FU
Reduction in recent alcohol use.	**Biological:** Measured by the presence of ethyl glucuronide (EtG), a breakdown product of ethanol, using an EtG urine test. Alcohol urine tests measure past 10 days of use. **Self-report:** Consumption days, problematic drinking days (4+ for women; 5+ men), combining alcohol with other drugs.	Baseline, 3 months, 6 months FU
Reduction in gender-based violence. FG	**Self-report:** Relationship violence, physical abuse, sexual abuse, and recent victimization measured by modified World Health Organization and RRBA items.	Baseline, 3 months, and 6 months FU
Reduction in sexual risk: Increased condom and contraception use, decreased number of partners or concurrent partners, decrease in impaired sexual encounters.	**Self-report:** Last sex condom use, number of sex partners, concurrent sexual partners and sex trading partners, AOD use prior to sex, and use and types of contraception asF measured by the PRBA+.	Baseline, 3 months, and 6 months FU
Greater equity in traditional attitudes about gender roles, empowerment, and assertiveness;	**Self-report:** Attitudes about traditional gender roles, empowerment and assertiveness will be measured by the GIS and the PRBA+.	Baseline, 3 months, and 6 months FU
Increased communication between partners regarding testing, HIV, and STI/STD status; other partners; sexual negotiation; and condom use.	**Self-report:** Communication with partner about testing, HIV and STI/STD status, other partners, sexual negotiation and condom use as measured by the PRBA+.	Baseline, 3 months, and 6 months FU
**Aim 3: The Couples Health CoOp Plus proposes to increase both partners’ PrEP/ART initiation and adherence. fgAim 4: Examine the interaction of the stigma awareness workshops and Couples Health CoOp Plus intervention on increased PrEP and ART initiation, retention, and adherence for both partners.**		
FGEvidence of ART or PrEP initiation.	**Medical Record Review:** ART/PrEP initiation and HIV status as documented by medical record.	6 months FU
Increased biological evidence of ART or PrEP adherence.	**Biological:** ART adherence will be measured using viral load or cluster of differentiation 4 (CD4) count measures recorded in the participant’s medical record. Threshold for adherence will be cluster of differentiation 4 (CD4) count (more than 200) and HIV viral load (less than 50 copies per ml). Tenofovir Diphosphate (TFV-DP) and FTC Triphosphate (FTC-TP) concentrations in dried blood spots (DBS) with drug concentrations of greater than 700 femtomoles (fmol)/liters(L) will be considered adherent to PrEP.	6 months FU
Increased reporting ART or PrEP adherence.	**Self-report:** Past 30-day use, missed doses, time in weeks, and patterns of use of ART or PrEP as measured by the RRBA/PRBA+.	3 months, and 6 months FU
Increased evidence ART or PrEP adherence; Increased persistence and reduced discontinuation.	**Medical Record Review:** Refills, number of months on ART/PrEP since initiation, and follow-up visit attendance as recorded in clinic medical records.	6 months FU
**Note:** AOD, alcohol and other drugs; ART, antiretroviral therapy; ASSIST, Alcohol, Smoking and Substance Involvement Screening Test; GIS, Gender Ideology Scale; PRBA+, Pretoria Risk Behavior Assessment; PrEP, pre-exposure prophylaxis; RRBA, Revised Risk Behavior Assessment; SRH, sexual and reproductive health; STI/STD, sexually transmitted infection/disease.		

### Statistical analysis plan and hypotheses

We will examine the distribution, frequency, and missing values in each variable using frequency tables for categorical variables and univariate distributions for continuous variables. We will compare data across the arms to examine the balance of baseline demographic and clinic characteristics using chi-square tests and t-tests or their nonparametric alternatives, as appropriate. Next, we will examine the association between demographics, clinic characteristics, primary outcomes, and any important mediators and moderators to determine which variables will be entered in the generalized linear models (GLM). We will use a GLM with generalized estimating equations (GEE) to test the relative efficacy of the interventions on the primary outcomes. GEE allows one to adjust for clustering and accommodates multiple types of outcomes (dichotomous, continuous, ordinal) and dyadic data analysis. We will use the couple as the unit of analysis for ART/PrEP initiation and adherence. First, we will fit a logit model that regresses the dependent variable on the membership in the arms and visit, and an interaction between visit and arm. Second, if descriptive analysis suggests there are significant differences in demographics or characteristics of clinics across the arms, those variables will be added into the model as covariates. We will test ART/PrEP initiation and adherence across two arms at a time by estimating contrasts at each time point to test both the hypotheses that the CHC+ increases ART/PrEP initiation and adherence. We will use an identical process to estimate the impact of the interventions on AOD use, GBV, sexual risk-taking, communication, and gender norms. The distribution in the GLM will be modified to be multinomial for ordinal outcomes, binary for dichotomous outcomes, and continuous or Poisson for continuous or count outcomes, respectively. The unit of analysis for outcomes (e.g., condomless sex) will be at the couple level and/or individual level, when appropriate, which the study team has done in previous couples-based analyses [15]. Analyses will be performed for the 3-month and 6-month follow-up time periods to determine if there are differential effects over time on ART/PrEP initiation and adherence, and at the 6-month time period for secondary outcomes.

### Data and safety monitoring plan

To prioritize confidentiality, each participant in the study is assigned a unique alphanumeric study participant identification number (PID), which serves as the sole link between the behavioral and biological data and the identifying information collected. Safeguarding the privacy of participants is of utmost importance. Data collected through ACASI are transmitted securely to a central server at RTI International in the USA. Personally identifiable information (PII) is stored separately and is accessible only to authorized study staff. To ensure the highest level of security, deidentified DBS samples are stored and shipped for analysis to certified laboratories that adhere to strict data management protocols, maintaining confidentiality and complying with ethical standards. The data collection process is carried out by highly trained staff from the local community, who establish rapport with the study participants to foster trust and obtain the most accurate data possible. All staff members sign confidentiality agreements and undergo comprehensive training on the study’s Quality Assurance Protocol and Quality Management Plan (QMP). A robust quality assurance plan has been developed and regular process monitoring at each step is conducted to review files and ensure fidelity of each intervention. Training is conducted on the manualized field procedures and a post training manual test and regular booster training are also conducted. To maintain oversight and ensure efficient operations, the Multiple Principal Investigators, other members of the research team, and the field staff receive daily field activity reports. This allows for continuous monitoring of progress and facilitates prompt attention to any issues that may arise.

#### Reporting of adverse events

Procedures have been put in place to address adverse events (AEs) or serious adverse events (SAEs), such as improper disclosure of information or mental or emotional discomfort, respectively. As specified in our Data and Safety Monitoring Plan (DSMP), SAEs are reported immediately to the South African Multiple Principal Investigator or Medical Director, who will contact the US-based Contact Principal Investigator. Based on advice from the aforementioned project staff, the field staff will make appropriate referrals to medical, counseling, and/or other health services. SAEs and Incident Reports will be reviewed immediately. SAEs will be reported within 24 hours of their occurrence by the Principal Investigator to the Project Officer, ethics committee, and the Chair of the Data and Safety Monitoring Board (DSMB). A DSMP ensures that procedures have been set in place to safeguard the security, validity, and integrity of study data, and that study staff are trained on the policies and procedures for data management according to the Quality Assurance Protocol. The DSMP also outlines the data analysis plan, including preliminary analyses of data for quality assurance and to track the progress of the study.

### Dissemination plan

The study findings will be disseminated to communities and stakeholders through the PAB, CCB, program newsletters, peer-reviewed journal articles, conference presentations, and targeted channels. This study team also has had success in conducting policy and impact forums with community members and the government to further disseminate study findings. This forum will provide the opportunity for conversation with South African government officials on how this intervention could be scaled up.

### Protocol amendments

The study adheres to ethical guidelines, obtains informed consent, maintains participant confidentiality, and provides referrals for necessary support services. All study activities have been reviewed and approved by relevant ethical review boards. Since receiving approval to conduct the randomized trial, we amended the protocol to improve the study design and procedure.

As the study was delayed in the initial commencement of the trial, we removed the 12-month follow-up, prior to the experimental phase. We also removed the 9-month follow-up after the trial commenced because of timeline concerns related to social unrest in the study communities.

## Discussion

South Africa faces numerous challenges, with disruptions in electricity, water shortages, ongoing strikes, and violence. Since the beginning of the COVID-19 pandemic, healthcare providers have been stretched and in 2024 public health services budgets have also been cut, further challenging the most economically disadvantaged communities and key populations in receiving necessary services. Community factors—including high rates of poverty and people’s continued use of AODs, which also intersect with condomless sexual risk behavior, gender-based violence, and the high burden of HIV among young women and their partners—reinforce the need for working with young couples and within their communities. This protocol describes the Couples Health CoOp Plus (CHC+) study, which aims to address these intersecting epidemics by integrating ART/PrEP initiation and adherence within the existing CHC model and by conducting community stigma awareness workshops. By adopting a multilevel social ecological framework, the study seeks to enhance HIV prevention efforts, improve treatment outcomes, and alleviate the burden of HIV in this key population and raise community sensitivity. The study findings have the potential to guide future interventions and contribute to ending the HIV epidemic in South Africa.

## Supporting information

S1 Checklist SPIRIT 2013 checklist: Recommended items to address in a clinical trial protocol and related documents*. (PDF)

S1 File (DOCX)

## Abbreviations

ACASI: audio computer-assisted self-interviewing
ART: antiretroviral therapy
ASSIST: Alcohol, Smoking and Substance Involvement Screening Test
CCB: Community Collaborative Board
DBS: dried blood spots
DSMP: Data and Safety Monitoring Plan
ETG: ethyl glucuronide
FTC-TP: FTC Triphosphate
HTS: HIV testing services
PAB: peer advisory board
PII: personally identifiable information
PrEP: Pre-exposure prophylaxis
QMP: quality management plan
RRBA: Revised Risk Behavior Assessment
SAE: serious adverse event
SRH: sexual and reproductive health
STD or STI: sexually transmitted disease or infection
