# Supplementary material for: Multilevel strategies to end HIV for young couples in Cape Town: Study protocol for a cluster randomized trial
Source: PLoS One. 2024 Jun 7;19(6):e0305056. doi: 10.1371/journal.pone.0305056 (PMC11161046; doi:10.1371/journal.pone.0305056)
Supplement: S1 File — (DOCX) [file pone.0305056.s002.docx]

**RESEARCH PROPOSAL**

**12^th^ November 2021; Revised: 11 July 2022; Revised: 20 January 2023**

**Version 7.0**

| Multilevel Strategies & Tailored HIV Prevention & Care for Young Couples who use Alcohol & Other Drugs Across Cape Town: Couples Health CoOp Plus [CHC+]  EC031-8/2020 |
| --- |

**STUDY PRINCIPAL INVESTIGATORS**

Contact PI (USA): **Dr Wendee Wechsberg (PhD)**

RTI Global Gender Center and Substance Use, Gender, and Applied Research

RTI International

Research Triangle Park

North Carolina

United States

27709-2194

Email: [wmw@rti.org](mailto:wmw@rti.org)

Multiple PI (USA): **Dr Felicia Browne (ScD)**

Substance Use, Gender, and Applied Research

RTI International

Research Triangle Park

North Carolina

United States

27709-2194

Email: [fbrowne@rti.org](mailto:fbrowne@rti.org)

Multiple PI (SA): **Dr Tara Carney (PhD)**

Alcohol, Tobacco and Other Drug Use Research Unit

South African Medical Research Council

Tygerberg

South Africa

7505

Department of Psychiatry and Mental Health

University of Cape Town

Rondebosch

7701

South Africa

Email: [tara.carney@mrc.ac.za](mailto:tara.carney@mrc.ac.za)

**Study Sites***:

***South African Medical Research Council of South Africa (SAMRC)***

Alcohol, Tobacco and Other Drug Research Unit

Tygerberg, South Africa 7505

***SAMRC Delft Office***

Brentwood Park,

Driftsands

7100

***RTI International***

Substance Use, Gender, and Applied Research (SUGAR) Program

3040 East Cornwallis Road

P.O. Box 12194

Research Triangle Park NC 27709-2194

United States

**OTHER KEY PERSONNEL***

| **Name** | **Institution** | **Role** | **Email address** |
| --- | --- | --- | --- |
| **Dr Catherine Orrell** | Desmond Tutu Health Foundation/UCT | Co-Investigator/  Medical Director | catherine.orrell@hiv-research.org.za |
| **Dr Laura Nyblade** | RTI International | Co-Investigator | lnyblade@rti.org |
| **Dr William Zule** | RTI International | Co-Investigator | zule@rti.org |
| **Dr Courtney Bonner** | RTI International | Co-Investigator | cpbonner@rti.org |
| **Jacqueline Ndirangu** | RTI International | Project Director | jndirangu@rti.org |
|  | SAMRC | Field Site Supervisor |  |

*****Consultants include Dr Bronwyn Myers (SAMRC/Curtin University) and Dr Dvora Joseph Davey (UCLA/UCT)

**Funded by: National Institute on Drug Abuse (NIDA), Division of National Institutes of Health (NIH). The proposed study is an R01 trial and has been through extensive scientific peer review prior to the study award.**

**PROJECT KEYWORDS**

Alcohol and drug use, stigma, couples, HIV services, PrEP, ART

***ART and PrEP to be provided at relevant City of Cape Town healthcare clinics. City of Cape Town is currently rolling out their PrEP implementation plan.**

**CONTENTS**

[DECLARATION 3](#_Toc96953984)

[1. INTRODUCTION AND RATIONALE 5](#_Toc96953985)

[2. STUDY AIM 7](#_Toc96953986)

[3. STUDY INNOVATION AND APPROACH 7](#_Toc96953987)

[4. METHODS 11](#_Toc96953988)

[5. ETHICAL CONSIDERATIONS 16](#_Toc96953989)

[6. REFERENCES 21](#_Toc96953990)

# INTRODUCTION AND RATIONALE

***Reaching Young Women and Their Primary Sex Partners with Alcohol and Other Drug (AOD) use continue to impact HIV transmission in South Africa***

The intersectional epidemics of alcohol and other drug (AOD) use^[1]^, sexual risk behaviour, and gender-based

violence (GBV)^[2-4]^ in South Africa contribute to an HIV syndemic, especially among young women. This

syndemic exacerbates the disease burden, with HIV prevalence among women increasing across the lifespan from 6% to 16% to 28% among 15 to 19, 20 to 24, and 25 to 29 years of age, respectively^[5]^. Our studies have found

high rates of AOD use. Because a majority of HIV transmission in South Africa continues to occur through

heterosexual contact, reaching these young women and their primary male sex partners for HIV testing services (HTS) and linkage to ART (antiretroviral therapy) and PrEP (pre-exposure prophylaxis) is imperative.

While most prior research has focused on the relationship between age-disparate (partners 5 or more years older) or intergenerational sexual relationships (partners 10 or more years older) among young women and HIV transmission^[6, 7]^, many women in young adulthood have primary male partners who are closer to their age ^[8, 9]^ and who may be at increased risk of HIV if 1) they are having sex with concurrent male partners or 2) they have had previous partners who are older^[9, 10]^, as they are more likely to be living with HIV, and less likely to be on ART as they are less likely to receive health services^[10-12]^. Because adolescent girls and young women (AGYW) seroconvert 5 to 7 years earlier than their male peers, these male partners are in turn at increased risk for HIV from their same-age female partners if they are HIV positive^[5]^. Male partners need to be engaged in the HIV continuum to support their own and their female partners’ HIV prevention, as partners may help reduce HIV risk by providing support to each other for HIV testing, treatment, and medication adherence^[13-17]^.

It is also essential to address heavy AOD use among young women and their partners, which increases the likelihood of outside sex partners, gender-based violence and impaired condomless sex^[13, 18, 19]^ and reduces adherence to ART, which can increase viremia and the risk of onward transmission. South Africa has high levels of alcohol consumption, increasing levels of opiate use^[20]^, and in Cape Town – especially high rates of methamphetamine use (known as “tik”). AOD use also reduces personal agency to negotiate condom use. These individual and relational challenges may impede access to health services and be barriers to achieving the 90-90-90 goals as related to HIV^[21]^, with adherence to treatment having a positive impact on viral load.

***Strategies to Address HIV***

Developing effective strategies for ensuring that the virus remains undetectable and therefore untransmittable

(U=U) is the new frontier for ending the HIV epidemic^[22]^. HIV treatment as prevention^[23]^ is the leading strategy

for reducing HIV infections. South Africa has successfully expanded its HIV testing and ART program. However, more effective interventions are needed to reduce incidence among young people (30 years old or younger), to reach South Africa’s goal of a 63% reduction in new HIV infections by 2022^[24]^. South Africa has approved oral PrEP to prevent HIV infection^[25]^. Yet, ART and PrEP availability cannot curb the epidemic without sufficient initiation and adherence, which requires addressing contextual issues. As mentioned earlier, the highest HIV incidence in South Africa is among women in young adulthood ^[5]^, some of whom are in relationships with a primary partner in which AOD use, condomless sex, and outside sex partners are common^[26, 27]^. Consequently, a comprehensive biobehavioural approach focused on reaching young women and their main sex partners is needed to address these intersectional epidemics. Empowering couples who use AODs with the knowledge and skills to reduce their risk behaviours and improve their relationship has been effective.

***HIV interventions can be more effective when both partners are involved***

Couples-based interventions are efficacious for ART adherence ^[15]^ and show promise to improve PrEP adherence ^[28-31]^. Couples-based interventions in Sub-Saharan Africa have the potential to avert up to 66% of HIV cases,^[13, 32]^ and those that promote strategies such as ART, PrEP, condom use, communication skills, and mutual support may lead to better outcomes^[15, 28, 30, 33]^. In 2001, we began developing and testing behavioural interventions in South Africa to reduce AOD use, sexual risk, and GBV among women who use AODs through the Women’s Health CoOp (WHC), developed by Dr. Wechsberg (contact PI) ^[3, 34, 35]^. Over the years, we expanded our interventions to include adolescent girls and young women (AGYW) and couples ^[13, 27, 36, 37]^, with the HIV epidemic affecting young women disproportionately^[5]^. As the science advanced, we incorporated biomedical advances (i.e., antiretroviral therapy [ART] and pre-exposure prophylaxis [PrEP]) as a status neutral approach. In 2019, in other parts of South Africa, an existing study implemented by Dr. Wechsberg, and the study team began to intervene at the structural level by engaging clinic staff in stigma-education training to reduce barriers for AGYW accessing sexual and reproductive health (SRH) care and PrEP. However, men have been left out of this equation and gaps exist with young couples who use AODs for a comprehensive biobehavioural approach, which we hope to address in the revised multilevel strategy.

***The Existing Couples Health CoOp***

The Couples Health CoOp (CHC) is an empowerment-based intervention developed for South African couples—that addresses the syndemic of AOD use, GBV, and sexual risk among couples at risk for HIV^[38]^. The existing CHC is grounded in Social Cognitive Theory^[39]^ and promotes prevention strategies that address the relational context of equality in which sexual risk takes place. Consequently, the CHC was developed for couples who use AODs and engage in condomless sex to work together to increase knowledge, skills, and agency to reduce AOD use, GBV, and sexual risk within their relationship. The CHC was adapted from the WHC^[34, 40]^, an evidence-based, women-focused HIV intervention that focuses on the intersection between substance use, sexual risk and gender-based violence, and Project Connect^[14]^, an efficacious couples-based HIV intervention. One of the salient adaptations of the WHC when adapting it for South Africa 20 years ago was the inclusion of a module that focused on addressing violence and victimization. In a 3-arm trial (n=300 couples; mean age: 24 [women]; 26 [men]), the CHC was efficacious in increasing condom use (Cohen’s d=0.23), improving gender norms (Cohen’s d=1.13), decreasing heavy alcohol use among men (Cohen’s d=0.42), and decreasing HIV incidence among

women (Cohen’s d=0.31)^[13, 41]^. In dyadic analyses, both partners in the CHC arm were significantly (*p* < 0.001)

more likely to report positive gender norms^[41]^. All three PIs collaborated on the development of the previous CHC intervention, which was led by the contact PI (Wechsberg).

Four years later, focus group discussions (FGDs) were conducted with a randomly selected sample of couples who participated in the CHC found that women reported “*reduced* *fighting”*; whereas men reported “*being faithful and more loving*.” Participants suggested that communication was key and that the intervention should be continued as “*there is often sharing of (sex) partners*" and HIV-positive individuals still experienced “*challenges to adhering to HIV drugs, especially if they are still using alcohol*.”^[42]^ Although the CHC demonstrated efficacy in reducing HIV risk among couples, it did not educate about ART or PrEP uptake and adherence. The logical next step was to **modify** the evidence based CHC as a biobehavioural intervention to promote ART/PrEP initiation and adherence in this study for young women and their main partners (the Couples Health CoOp Plus [CHC+]) with information about and linkage to PrEP and ART in preparation for our randomized trial, which has now been completed. Following feedback from young couples, our Peer Advisory Board and Community Advisory Board and consequent modification, the CHC+ now includes information on sexual and reproductive health, being status neutral, U=U and ART and PrEP. Please see Study Material (**Exhibit 16**) for a list of the revised contents of CHC+, as well as an example of new slides which have been added on these topics.

***Existing structural barriers such as stigma hinder initiation and adherence of ART/PrEP among couples*** Stigmatizing attitudes and behaviours of providers may be a barrier for young women and their partners to access HIV and other SRH services, and reduce access to these services^[43-47]^.This includes the uptake and retention of PrEP and ART. Couples who use AODs, where one or both partners are living with HIV, and engage in sexual risk behaviours may face stigma at number of levels, including within their community. Efforts to reduce HIV risk must therefore address stigma, not just within healthcare settings, but also in the broader community.

where these couples are based. The Health Policy Project (HPP) HIV-Stigma and Discrimination Reduction-Training Curriculum (which we plan to use in our randomized trial) is based on a decade of implementation experience in 9 countries^[48-51]^ including Vietnam^[52]^, China^[53, 54]^, and Thailand^[55]^.The training address 3 key actionable drivers of HIV-related stigma: lack of understanding of stigma and discrimination; fear of workplace HIV transmission; and attitudes toward people living with HIV or engaging in high-risk behaviours, and is easily adaptable^[56, 57]^. The HPP led to the HP+, a Total Facility Approach Project aimed at addressing stigma among healthcare providers. The HP+ project showed large effect sizes related to reduced observed stigma toward youth and people with HIV in Ghana and Tanzania^[58]^. This curriculum has been adapted for clinics in South Africa as part of a current PrEP study (R01HD094629, PI: Wechsberg) for AGYW, the next step is to move out of clinics into communities. By ensuring that the issues that young men and young women in relationships face, including stigma at the community level around alcohol and other drug use and HIV (and treatment in terms of ART and treatment as prevention in terms of PrEP) need to be addressed. If community members’ beliefs, attitudes and behaviours are trained in stigma reduction, this may affect the support that these young people are able to obtain and may assist them in obtaining help for problems related to alcohol and drug use, and also access healthcare including ART and PrEP at clinics within their communities.

# STUDY AIM

The specific aims for the study are as follows:

**Aim 1.** To modify the CHC intervention to include ART/PrEP in a formative phase and with review from our Community Collaborative Board and a Peer Advisory Board *(this aim is complete, and the outline of the modified CHC intervention, referred to CHC+ is included in the enclosed exhibits of study materials)*

**Aim 2.** To evaluate the impact of a stigma reduction training ***and education*** on ***community members*** attitudes and behaviours toward young women and their primary male partner seeking HIV services (testing/ART/PrEP) and other sexual and reproductive health services in their local clinics at ***4- and 8-*** month follow up.

**Aim 3.** To test the ***efficacy of the CHC***+ to increase both partners’ PrEP/ART initiation and adherence (at 3 and 6 months), and reduce AOD use, sexual risk and GBV, and enhance positive gender norms and communication relative to HTS.

**Aim 4.** To examine through mixed methods the ***interaction of the stigma-reduction training and the CHC+*** on increased PrEP and ART initiation, retention, and adherence among young women and their primary partners.

# STUDY INNOVATION AND APPROACH

Innovation

***A new, adapted HIV prevention and care model***

**Figure 1 Adapted Integrated Conceptual Model**
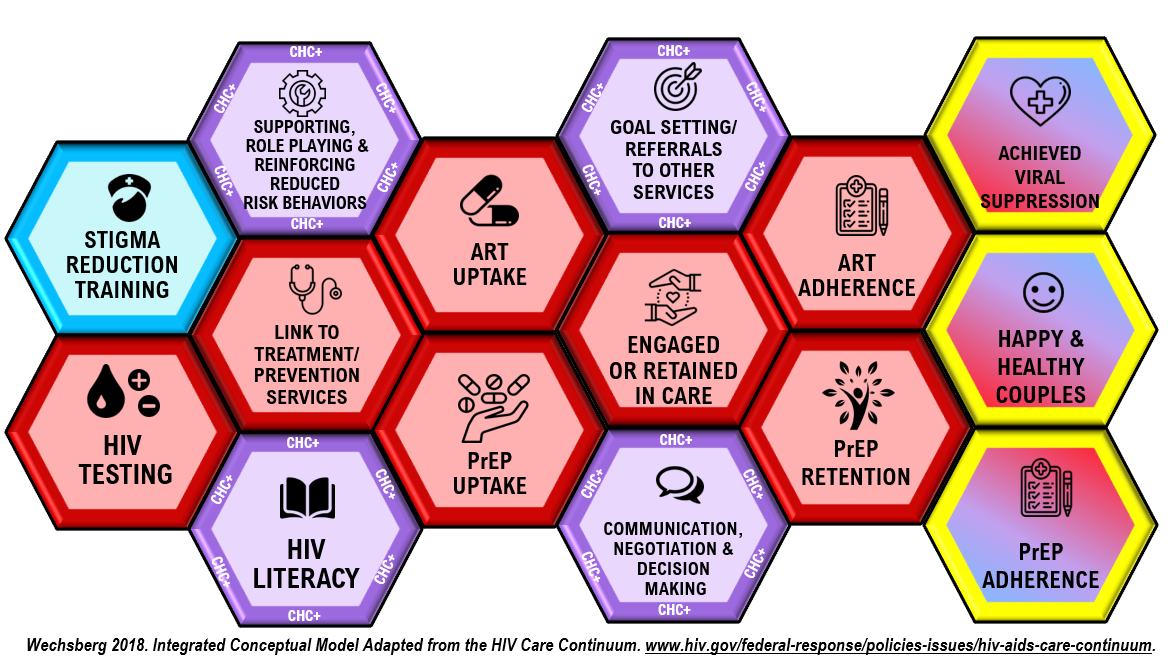


Building on our previous trials in South Africa, our innovation arises from scientific (i.e., biological) advances and identifying gaps in previous research (see ***Figure 1***). We will address discordant and concordant couples and combine the evidence-based (1) HIV status neutral approach for individuals, (2) behavioural intervention for couples, (3) clinic-level stigma-reduction training, and (4) PrEP/ART. Individuals within couples may be at different stages of the HIV continuum of care or prevention. Although combination prevention has been used to prevent HIV among serodiscordant couples,^[29,59-61]^ few studies have applied a status-neutral approach to prevent HIV among couples living with and/or at risk for HIV ^[13,62,63]^. This study proposes to reduce HIV risk among serodiscordant and concordant negative and positive couples who have outside partners, which addresses a missed opportunity. This new comprehensive biobehavioural approach 1) promotes ART/PrEP initiation and adherence, which helps reduce risk behaviour and supports couples together with the CHC+ intervention+ and 2) implements stigma-reduction training in catchment areas around clinics, which is essential to achieve the goal of viral suppression and no new cases. The Seek, Test, Treat and Retain (STTR) paradigm becomes more efficient reaching all.

***Stigma-reduction training within communities***

Clinic-level stigma and discrimination programs have been conducted to help reduce stigma toward younger populations; however, based on the findings of our formative work we suggest that stigma-reduction training be provided within communities surrounding healthcare clinics, as stigmatizing behaviour and attitudes can be enacted by family, friends and community members before individuals even come into contact with the healthcare system, and could act as a barrier to seeking treatment or prevention services. This adapted curriculum will also be further refined to include issues of young couples in Cape Town. Our study proposes to reduce community stigma to enhance initiation and adherence for ART/PrEP. This training will be supported by Peer Advisory Board (PAB) members and key community members for sustainability. This also supports the STTR paradigm.

Approach
*Multilevel social ecological framework*

The social ecological frame­work is based on the notion that behaviour occurs in a social and environmental context organized into recurring patterns of activity within life domains and structured environmental settings^[64]^. This framework has been advanced and developed in the fields of health promotion and behavioural medicine^[64, 65]^. More recently, this framework was adapted to HIV serodiscordant couples’ interventions. This conceptual framework has guided a number of couples-based approaches, including the proposed team’s research. This framework will guide the proposed study, and we will focus on the individual, couple, and structural levels as shown in Figure 2 below.

***Study Investigators and collaborations***

Our team reflects a long-time collaboration between the three MPIs across numerous trials. **Dr. Wechsberg,** contact MPI, has led HIV prevention trials in South Africa for 18 years with vulnerable women, couples, and young people and has been committed to HIV prevention for more than 30 years. She has clinical training, was a treatment director in the US and has many years of experience in addressing alcohol and drug use problems and expertise in young adulthood, in addition to PrEP and ART. **Dr. Browne**, MPI, was integral in the CHC’s development and has expertise in mixed methods research, including cluster randomized trials. She is an MPI on an R01 trial for young adults in clinics. **Dr.** **Carney**, the South African MPI, was a collaborator on the CHC and has been responsible for the operational direction of several studies in South Africa. The focus of her research is on alcohol and drug use, especially among young people and mental and physical health outcomes. Her training is in psychology, psychiatry, and mental health. They have assembled an outstanding international multidisciplinary team, including Drs. Nyblade (stigma reduction in clinics), Orrell (ART/PrEP), Joseph Davey (PrEP; couples), Zule (couples; AOD), Bonner (licensed psychologist with experience in stigma, AOD, mental health), and Ms. Ndirangu (10 years of global project management, including living in South Africa and managing projects in clinics). Dr. Myers, a clinical psychologist, is also a consultant on this study, with extensive clinical and research experience in mental health, including trauma and substance use. Previous research is shown in ***Table 1***.

| **Table 1 Previous Research Conducted by Members of the Proposed Investigative Team** |
| --- |
| ART/PrEP in South Africa. Research has shown that early ART initiation and retention is essential to HIV treatment and reducing transmission among key populations. PrEP research among key populations has found that PrEP knowledge is low and that sociocultural factors may serve as potential barriers to PrEP initiation. Alcohol use was a barrier to ART adherence among women, and young women were less likely to be adherent, but after a gender-focused intervention initiation and adherence increased (see letters in Section 9). |
| Stigma & Training. Research has demonstrated the effectiveness of the stigma-reduction curriculum & training to reduce stigma among health workers in multiple settings. This curriculum has been adapted in South Africa. An implementation study in Cape Town clinics found that stigma from clinic staff is a barrier to seeking health services for women who use AODs and there is a need for interventions to address this stigma. |
| Couples. The CHC in Cape Town was efficacious in reducing HIV risk & incidence (for women) among heavy alcohol-using men & their female partners. In dyadic analyses, partners in the CHC were more likely to report positive gender norms, and long-term follow-up found communication skills were sustained. A study in Southern Africa found among discordant couples, women were less likely to initiate ART if heavy drinkers. In addition, the CHC  was associated with an increase in women’s perceived level of control in their relationship, and the CHC and men’s intervention led to reduced reported  victimization within relationships. |
| Recruitment & Follow-up. We just completed recruitment of 500 young women for a prevention trial & 480 women for a treatment trial, demonstrating our ability to recruit this age group. In previous CoOp R01 studies with women & couples, follow-up at 6 & 12 months > 90%.[^8^](#_ENREF_8)^,^[^11^](#_ENREF_11) |

***Study process.*** Our process model with timeline is shown in ***Table 2.***

NOTE to reviewers: all formative work in preparation for the randomized trial has been completed. This was discussed in our previous submission and has therefore been excluded from this submission.

**Table 2.**

**Please note: Year 1’s activities (which we applied for previous scientific review for) have been completed.**


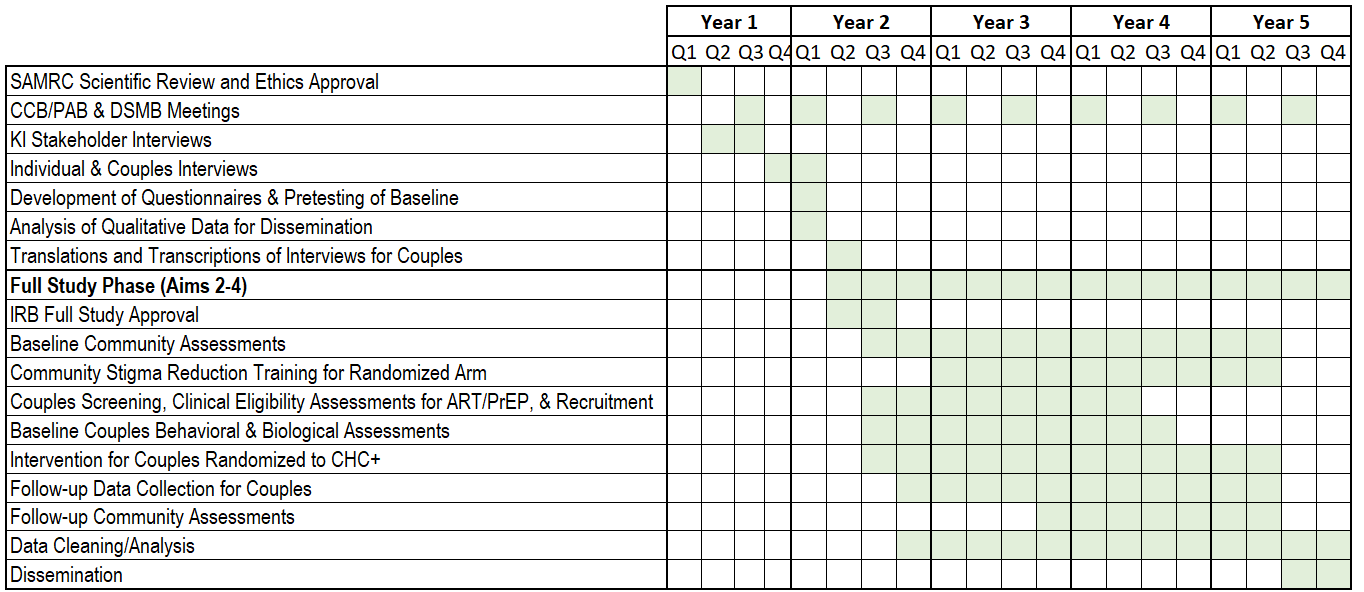


**Figure 1. Outline of Proposed CHC+ (New items in Bold)**


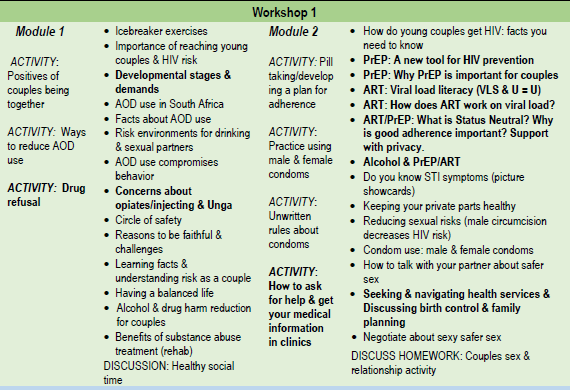


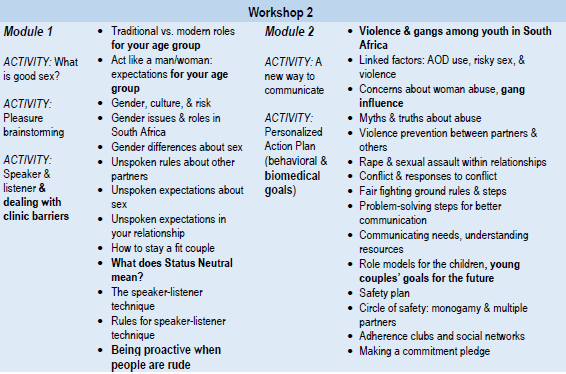


***Stigma-reduction curriculum, training, and instrumentation***

The stigma-reduction curriculum and training used in clinics for our ongoing NIH-funded study in Pretoria will be adopted for use in a community setting and reviewed with the Community Collaborative Board (CCB) and Peer Advisory Board (PAB) to be acceptable for the community setting in the Cape Town context. A brief community survey has already been developed and is ready for pretesting. Key aspects of the training, which will occur in the experimental phase, include (a) community stigma awareness training of people living with HIV, use of ART, PrEP for prevention of HIV and attitudes towards people who use alcohol and drugs. Selected modules will be modified from the existing clinic-based training to fit community stigma awareness and attitudes, as needed, to focus on aspects of stigma specific only to young women and men who engage in syndemic-related behaviours who live in their communities. We have developed the brief survey based on these constructs and are using adapted measures that have been previously used in a diversity of settings. These measures are now ready to be pretested and reviewed with the CCB and PAB. Co-I Dr. Nyblade will ensure that the stigma-reduction curriculum and survey are finalized for the experimental phase.

***Randomization***

City Health clinics within or adjacent to economically disadvantaged communities in the Cape Town area that have been selected for the government PrEP roll-out have been identified. After mapping these clinics, we will remove those clinics that will not be accessible to potential participants due to distance, for a total of 24 catchment areas or communities. As in previous studies, ^[13, 62, 63]^ we will create matched pairs and randomly select 12 pairs (a total of 24) from the list just before the trial commences. Then, the catchment areas around clinics within each pair will be randomized to receive the stigma-reduction training or no training. Finally, the catchment areas around these clinics within each pair will be randomized to receive either the HTS (HIV testing services) or CHC+ (with HTS). We will match on characteristics of the community to ensure equal numbers across study arms and the randomization of clinic catchment areas (instead of couples) will reduce contamination across study arms. See ***Figure 3*** for study design.

**Figure 3. Study Design**

# METHODS

**Young Couples**

***Study Awareness & Outreach***

Trained outreach staff in pairs (male and female), with assistance from the established Peer Advisory Board (PAB), will create awareness in the catchment areas surrounding the study clinics where young people generally congregate, visiting these areas during different days and times. The PAB will not work in their own community to reduce potential bias and to protect the privacy and confidentiality of potential participants. They will assist in posting recruitment flyers and interacting with young women and their partners. Field staff will use established street outreach methods, including mapping hotspots and communicating with owners of local establishments and shops, salons and centers, and posting recruitment flyers in communities surrounding the selected clinics as we have done in previous studies ^[13,27,40]^. We will also re-hire skilled and trained outreach staff from prior studies who reflect the demographics of the study communities and are multilingual. To support outreach efforts, we will obtain permission to post the **Recruitment Cards and Fliers (Exhibit 1a & Exhibit 1b)** at these hot spots and other locations including social media where the study population may frequent, and these materials will also be given to community contacts.

***Screening & Eligibility***

During the initial contact, verbal consent will be obtained to screen potential participants to determine eligibility. Screening will occur individually and privately (female staff screen the female partner and male staff screen the male partner). A screening appointment will be made if both partners are not present during outreach (defined as educating and creating awareness)—as screening has to occur simultaneously to determine eligibility (**Exhibit 2 Appointment Card for Screening with Partner**. Screening will be done using the **Quick Field Screener** only by project staff and not the PAB (see **Exhibit 3)**. Staff will then compare answers (away from the screened couple) to determine initial eligibility. Staff will have knowledge of the eligibility criteria; if one partner does not meet any of the criteria, the couple is ineligible. Ineligible couples will neither be told the reason(s) for ineligibility nor which partner(s) did not meet the criteria.

If they meet the preliminary eligibility and are interested, staff will accompany couples to the study field site in Delft or meet them at a private gazebo (these have been used successfully in previous studies) within the relevant healthcare clinic grounds, where they will undertake final eligibility determination, consenting, and intake assessments with study staff. We plan to create awareness of this study to young women, men, and couples in the community. The study team has successfully recruited women, men, and couples in Cape Town.

**To be eligible**, young women and their partners must each meet the following:

1. be between age 18 and 30;
2. been in a relationship with each other at least 6 months with the intention of staying together for the next year;
3. report condomless vaginal or anal sex with their partner in the past 3 months,
4. did not participate in previous Couples Health CoOp Plus study activities,
5. report the use of alcohol or drug use (e.g., marijuana, methamphetamine (tik), methaqualone, cocaine, or heroin) at least once a week for the past 3 months;
6. not currently on PrEP or ART (including as part of a study) or have not taken PrEP/ART in the past 3 months;
7. consent to urine drug and alcohol testing;
8. consent to sign release for medical records;
9. report not being on multidrug resistant TB (MDR-TB) medication; and
10. not pregnant and consent to pregnancy testing (for women)
11. At least one of the partners must report having condomless sex with someone other than their primary partner in the past 6 months, which will not be disclosed to their partner.

Couples will be excluded if one or both do not meet PrEP/ART clinical eligibility ^[67]^ from the clinic (e.g., for PrEP, have normal renal function via creatinine levels; for ART, normal kidney functioning). Excluded couples will not be counted as part of the total study sample (N=480 couples). Based on the contact PI’s current PrEP study with AGYW, we anticipate very few people to be excluded due to this reason.

For those eligible, basic contact information (e.g., phone numbers and nicknames) will be collected at this time in case the young couple cannot be scheduled for an appointment or fails to show up for their scheduled appointment. This information will be detached from the rest of the Screener and stored in a double-locked filing cabinet. Those who are screened eligible will be asked to bring some form of identification so the study staff can verify who they are at their first appointment.

Additionally, because of the eligibility requirement of non-pregnant status for women participants and knowledge of HIV status which will determine the ACASI questionnaire protocol, interested eligible potential participants must first consent to rapid testing for HIV and confirmation of pregnancy for eligibility. HIV Testing will be conducted according to the national guidelines [68], which include pretest and post-test counselling. Before conducting these tests, potential participants will be required to provide informed consent using the **Consent for HIV and Pregnancy Verification Testing (Exhibit 4)** and results will be recorded in the **Biological Verification of Eligibility Form (Exhibit 5)**. The field staff will conduct the screening and consenting. **If potential participants cannot be seen for intake (baseline) on the same day of their initial screening, they will not be tested until the day of their scheduled intake (as part of verification), to minimize instances of repeat testing.** Further, if potential participants are eligible based on the Quick Field Screener but cannot conduct the HIV and pregnancy test results on the same day and complete their intake appointment, they will be provided with an **Intake** **Appointment Card (Exhibit 6)** that has a reminder of their appointment date of their choice.

Rescreening of Participants: Potential participants will be rescreened separately using their original Quick Field Screener Form to ensure that they meet the study’s eligibility criteria on the day of enrolment. This will either be done at the field site, or in a private, secure space on the clinic grounds, where we will ensure that any information cannot be heard by other participants, clinic clients or staff and that this information will not be disclosed or shared with the clinic If a couple provides discordant responses to the screener questions that make one of them ineligible during this rescreening, then they will not be eligible to participate in this study. However, if the discordant responses do not affect eligibility, they will still be eligible to participate.

In the case that discordant responses result in ineligibility: To limit the potential for blame and/or violence between them (e.g., if one partner’s response is the reason why they are not eligible), couples will never be told exactly why they are not eligible for study participation. To ensure adherence to this protocol, project staff members will read separate scripts to ineligible and eligible participants, both of which are at the bottom of the screener.

***Informed Consenting Procedures***

Eligible individuals will be asked to provide informed consent for participation of the study prior to taking part in any study activity. There will be two types of consents- for couples in the intervention and for those not in the intervention (see **Exhibit 7a and 7b Couples Consent Forms) ***. Each individual will consent separately from their partner—all study activities during this appointment are separate. Study staff will be multilingual and written consent will be provided in either English or in the main languages used in the target communities, Afrikaans or isiXhosa. To obtain informed consent, the consent form in their language of choice will be read to potential participants, they will be given the opportunity to ask questions and have them answered, and to either initial or sign the consent form. The consent forms will include the following: a statement describing the extent to which the confidentiality of records identifying the participants will be maintained, including audio recordings, a statement that participation is voluntary, a statement that their involvement or early termination in the study will not affect their access to any services, an explanation of who to contact for answers to pertinent questions about the research and research participants’ rights, and who to contact for a research-related adverse situation such as an injury. Those who do not say in their own words what they are agreeing to and those who do not provide signed or initiated consent will not be enrolled. Participants will be reminded that they have the right to discontinue participation in the study at any point and to refuse to answer any specific questions if they choose to and will be given a card which contains their rights and contacts details if they have additional questions (see **Exhibit 8 Rights of Research Participants**).

Couples will also sign a **Release for Medical Records Form (Exhibit 9)** so that staff may access clinical staging results to prepare participants for PrEP and ART initiation, conduct follow-up surveillance for PrEP and ART (persistence and adherence), and other medical information and other health care information.

***PLEASE NOTE**: ONLY consents for baseline and intervention activities have been included here; follow-up consents will be submitted for ethics approval once the trial is underway.

***Intake (Baseline)***

After providing informed consent, a locator form will be completed, with the project staff asking the participant questions about where and how they can be contacted (see **Exhibit 10** for **Locator Form**). A picture of the participant will also be taken with a digital camera and stored with the Locator Form in a secure location. The digital file of the image will be immediately deleted after the photo has been printed out. The photo will be given back to the participant at their final follow-up appointment or else shredded.

Baseline assessments will then be self-administered to the participants via audio computer-assisted self-interviewing (ACASI; see **Exhibit 11 for Baseline Questionnaire**) in either English, Afrikaans, or isiXhosa. Couples will complete the ACASI separately and in a language of their choice. If intake appointments are conducted within gazebos on clinic grounds, ACASI also ensures that their responses cannot be overhead by their partners, other study participants or clinic staff. Trained project staff will be available to assist participants at any time. The study team has used this method successfully and it provides the advantage of having project staff help with the interpretation of questions to ensure comprehension and error checking done in real time. The software (Blaise®) for ACASI data collection has been used for many of our previous trials, including with couples in South Africa, and affords privacy and reduces social desirability bias. This baseline questionnaire is derived from components of the modification of the Revised Risk Behaviour Assessment (RRBA), which has been adapted in numerous studies in South Africa. The RRBA contains sections on sexual communication, STI symptoms, alcohol and other drug use, relationship equity and sexual control, economic dependence, personal agency, psychological distress, peers and social support, PrEP and ART knowledge, initiation and adherence, stigma and health services access and utilization.

**Biological Testing:** After completing the questionnaire, participants will undergo a urine drug screening to assess recent alcohol and drug use. If visits are to be conducted on clinic premises, we will arrange with the facility manager to use the clinic bathroom to collect urine, and the biological data tests will be put in ziplock bags and disposed of together with the lancets in small biohazard bins.  These tests will be repeated at follow-up assessments. Test results will be recorded on the **Biological Form** **for Intake (Exhibit 12)**.

**Referrals to ART/PrEP.** Participants will be referred to the clinic in their community area to be offered same-day ART or PrEP per recent South African National Guidelines ^[67]^. PrEP will be disseminated and monitored by the clinics based on national guidelines. Young women, discordant couples and people who use substances are currently priority populations for PrEP^[69]^. The City of Cape Town has a PrEP rollout plan which is currently being implemented, but we have discovered that PrEP may be inconsistently stocked in certain clinics. We will assess PrEP rollout in the study clinics and if needed, seek further donations. PrEP protocol includes repeated HTS upon refill that may not coincide with study HTS. To keep track of PrEP and ART initiation and refills, including PrEP persistence information the **PrEP Visit Record Form (Exhibit 13),** and **ART Visit Record Form** **(Exhibit 14)** will be completed throughout the study, respectively.

**Follow-up Appointments**

Behavioural and biological assessments of couple participants will be collected at 3- and 6--month follow-ups. The follow-up visit includes reconsenting; updating locator information; a follow-up questionnaire via ACASI; and biological testing for HIV, pregnancy, alcohol use and other recent drug use. Dried blood spots (DBS) also are collected on filter cards at these appointments from participants who report to be on PrEP or ART. Participants will be informed of the DBS procedures during consent the process including shipping, storage, and destruction of the blood samples. New consents will be signed at follow-up study visits before any study activities commence (**Exhibit 26 for 3-month follow-up and Exhibit 26b for 6-month follow-up**). As with the baseline appointment, assessments will then be self-administered to the participants via audio computer-assisted self-interviewing (ACASI) (**Exhibit 27**). Follow-up questions will include if participants used ART or PrEP in the previous 3 months, if they are currently using one of these medications and how often they took this medication, and plans to use this medication going forward. Participants will also undergo biological testing at follow-up appointments to assess alcohol and drug use as mentioned above, HIV testing if participants’ HIV test results were negative at baseline. In addition, we will perform dried blood spot tests as described below (**Exhibit 28**). Follow-up study visits will occur at 3- and 6--months post enrolment, regardless of when PrEP or ART is initiated or when a participants’ clinic visits take place.

As mentioned above at follow-up, participants will self-report initiation and adherence of PrEP or ART, if any. Given the need to validate these self-report measures, we will also collect biological measures of adherence for PrEP and ART through dried blood spots (DBS) and viral load suppression data and CD4 count from the National Health Laboratory Services in South Africa. This will always follow the activities that have been set out in the study’s DBS standard operating procedures (**Exhibit 29**). DBS specimens to assess levels of ART and PrEP drugs will be safely stored at the project site in Delft. Only upon signing an MTA (Material Transfer Agreement) between the provider (SAMRC) and recipient (RTI: Contact person is the project director Jackie Ndirangu), as well as obtaining the appropriate export and import permits (from the Centers for Disease Control and Prevention in the USA), will any blood samples be shipped for analysis to a laboratory at the University of North Carolina where the contact PI (Wechsberg) is also an associate member of staff. This MTA has been drafted and is currently being revised based on suggestions made by the SAMRC Legal Department. DBS samples will be stored in a freezer onsite (≤ -20°C) and shipped within 3 months. We also will validate HIV status and monitor any seroconversions by conducting HIV testing among HIV-negative participants at follow-up and refer people who test positive for HIV for ART. Clinics will assess medication resistance according to national guidelines.

Through the signed Medical Release of Medical Records Form provided at intake from all participants, we will collect patient information on ART and PrEP initiation and persistence throughout the study period from the clinics—and these data will be anchored to the follow-up time periods. This information will be completed in the aforementioned PrEP and ART Visit forms. As part of the PrEP protocol, the clinics will test for pregnancy for those who adopt PrEP. Women considered at risk may remain on PrEP if they test positive for pregnancy based on patient-clinician decision. ART/PrEP retention will be measured by questions regarding whether and how often they have attended a medical visit with an ART/PrEP provider in the past 3 months, and if this visit included laboratory results.

Referrals. We will also offer referrals for participants who self-report symptoms of STIs, pregnancy (at follow-up), or psychological symptoms consistent with a psychological disorder or suicidality. Additionally, should a participant disclose past or current physical or sexual victimization, they will be referred for additional counselling and support. Data on specific types of referrals, services, and duration of services received will be collected at follow-up by asking the participant and requesting clinic records (**see Referral Form (Exhibit 9) and Resource Guide (Exhibit 15)**)**.**

***Couples Health CoOp PLUS (CHC+) Workshops***

For couples enroled in the arm receiving the CHC+, the intervention will be facilitated at a community centre, library, or another location by trained project staff members. The CHC+ has been adapted from the evidence based CHC intervention to include a biobehavioural component to promote ART/PrEP initiation and adherence for young women and their main partners with information about and linkage to PrEP and ART. The CHC+ intervention is a 2-session, 4-module, 2-hour program that increases knowledge, skills, and agency to remain healthy as a couple, reduce GBV, substance use, and sexual risk to reduce HIV incidence, and communication (see **Exhibit 16 for Workshop Modules**) and are provided with a handbook of information as supplementary material (**Exhibit 17 CHC+ Handbook**) and risk reduction materials—e.g., male (external) and female (internal) condoms. Participants of these workshops will be asked to fill in a **Participant Satisfaction Form** to give us feedback on the intervention (see **Exhibit 18**).

***PrEP and ART Navigation***. PrEP and ART navigation, which occurs through phone calls and text messaging, will provide couples in the CHC+ intervention arms who are on PrEP and ART with much-needed support, in particular the first one to two weeks of PrEP initiation where many participants may experience minor side effects that often lead to PrEP discontinuation. Study staff work with participants to develop feasible plans that can support the daily use of PrEP and ART adherence strategies, social support, and addressing other concerns especially about PrEP, which is a novel HIV prevention method. Participants are also reminded about their upcoming refill and check-up appointments. This navigation occurs throughout the study duration as participants are due for their refills.

**Communities Stigma Assessment and Training**

We plan to recruit up to 97 individuals from each of the 24 communities in catchment areas of the clinics selected for this study (See **Figure 2: Research Design**). As part of the evaluation of the impact of community stigma reduction training, we will collect baseline data prior to the training workshops in the communities (all 24 communities - trained and untrained) using the **Community Questionnaire** (**Exhibit 19**). Community members participating in the baseline data collection will provide verbal and written consent to complete assessment (see **Exhibit 20a** **Community Stigma Survey Consent** for non-training and **Exhibit 20b Community Health Survey and Trainings Consent** for training communities). Participants of these workshops will be asked to fill in a Community Workshop Participant Satisfaction Form to give feedback on the stigma reduction workshops (see **Exhibit 21**).

We will conduct repeated surveys in the same communities 4- and 8-month after the initial community surveys were conducted. We will not request the inclusion of the same participants; therefore, this will be considered a more cross-sectional design to determine stigma reduction and awareness from the workshop and possible dissemination in a natural history effect. However, we will ask those who complete surveys at 4- and 8-months if they completed the original one so that we can document how many individuals naturally participate in repeat activities. In addition to asking questions that measure observed community stigma, 4- and 8-month repeated surveys will also include whether participants had completed this survey at a previous time point and attended any of the community workshop **(Exhibit 30).** The community surveys will be a brief paper-and-pencil, self-administered assessment similar to the one adapted from the stigma-reduction evaluation tools. To limit issues of social desirability, we also ask participants to not put their name on their survey and once their survey is completed, place it in a locked box. This method has also been implemented by Co-I Dr. Nyblade in previous community-based stigma studies.

***Community Stigma-reduction Training Workshops***

The stigma reduction training workshops will be delivered after but on the same day as the brief survey in communities randomized to receive this training (N= 12). These workshops will be conducted in settings such as community library workrooms, sports halls, or temporary tented areas outside an agreed space within the community following COVID restrictions **and precautions**. Ms. Ndirangu (Project Director) and along with an experienced project staff member, who are currently implementing a stigma-reduction curriculum in Pretoria, will serve as the key trainers, with oversight from Co-I Dr. Nyblade. Large campaigns will need to be held to inform people that live in these communities about the stigma reduction training. These workshops will occur ***over two sessions*** for an hour each in the randomized communities, a total of 2 hours will be designated including time to **consent, complete the survey, workshop, and the provision of refreshments after the workshop**. Childcare will be provided as needed, and these workshops will be conducted in the evening and/or over weekends.

***Data management***. Data management for the ACASI data collected on laptops will be conducted by RTI in the US using a standard system to transmit data via the Internet by the study staff based at the SAMRC at the end of each day from the laptops. This system tracks data that have been successfully transmitted and automatically deletes data from the laptops once the data are transmitted, they will be kept on a server at RTI and backed up on a separate password-protected server. The data manager will clean the data and prepare weekly reports during data collection. Forms containing personally identifiable information (PII) (e.g., locator and consent forms) will not be entered or transmitted to RTI and will be stored in double- locked files at the study site with access restricted to study staff. Unique alphanumeric identifiers will be used to link across records from the same individual. For any data collected via paper-and-pencil, it will be password protected and stored on laptops which will be double locked at the field site at the SAMRC.

DBS samples will be stored in a freezer at the SAMRC and then shipped to certified laboratory either in South Africa or at the University of North Carolina (UNC), Chapel Hill in the US for quantification of drug concentrations for PrEP. If at all possible, DBS specimens that will be used to measure ART levels will be sent to a certified laboratory such as Global Laboratories which meets all of the National Department of Health criteria for material transport and storage. The DBS will be identified by study ID, without participant names, to ensure confidentiality. Following completion of every run, the instrument data and result tables are copied to the laboratory’s secure network for storage. Backups will be performed nightly. Hardcopies of test results will be kept at the laboratory, filed by sample set name. Test results will be sent to the RTI in batches and stored on the secure project server at RTI. Any unused DBS samples will be destroyed at the completion of the study and will not be used for future research studies.

***Quality assurance, process measures, and fidelity****.* A quality assurance plan and manual will be developed for field activities and all staff will complete a test of comprehension before study involvement along with South African Good Clinical Practice, and National Institutes of Health (NIH) training and certification for good clinical practice in social and behavioural research. If PAB members assist with recruitment and marketing, they will complete a modified training. As part of process monitoring, all files will be reviewed at the end of each day. The project manager will use a form to observe or record the CHC+ the workshops for fidelity to content, exercises, and delivery, if all study participants in the applicable session agree to this. It is optional for participants to agree to the recording of workshops, as is listed in the study consent forms.

***Post-trial in-depth interviews to elucidate findings.*** After trial completion, a random sample of 40 CHC+ participants stratified by both CHC+ study arms, gender, and HIV status will be asked to participate in in-depth interviews to assess (1) barriers and facilitators to PrEP/ART initiation and adherence, including whether partners played a role, (2) experiences around stigma, (3) CHC+ experience, including what parts were most and least helpful for initiation and retention, whether they used any of the skills and techniques outside of the workshop with their partner, and whether they are still using any of the communication and empowerment strategies, and (4) changes in risky behaviour and gender norms within their relationship—for example, how they communicate and support each other. Interviewers and interviewees will be matched on language and gender. Analyses for interviews will be the same as for the FGDs in the formative phase with ATLAS.ti or another comparable qualitative software programme.

# ETHICAL CONSIDERATIONS

**Potential risks and benefits for participants**

This study presents no greater than minimal risk to any participants and adequate provisions will be made for soliciting the consent of the participants. However, there are five potential risks for participants: (1) possible disclosure of confidential information; (2) mental discomfort associated with gaining knowledge of biological test results and/or issues raised during ACASI or intervention sessions; (3) psychological, social, and legal ramifications of a positive HIV test result; (4) possible violent reactions to practicing risk reduction behaviours; and (5) side effects from taking PrEP or ART offered at the clinics but monitored by our field staff. Alleviation of these risks is discussed below:

***Benefits***

The potential individual benefits offered to study participants outweigh the risks associated with participation in this study. One potential individual benefit for participants from the study includes knowledge of one’s HIV status and subsequent access to prevention or treatment in cases where participants test positive for HIV. Currently, in South Africa same day PrEP and ART initiation (screen/test and start/treat) helps reduce barriers to uptake. Additionally, participants who take part in the CHC+ condition will also receive an intervention that has been adapted from a “best-evidence” HIV behavioural prevention intervention that teaches couples HIV prevention strategies. Previous studies in South Africa have demonstrated that the couples’ intervention reduces AOD use, increases condom use with main partner, and increases communication. As these factors relate to HIV risk, the knowledge and skills gained by participants may lead them to modify behaviours and adhere to their ART/PrEP regimen and so maintain U=U.

**Risk Mitigation Plan**

***Staff Training (including human subjects training) and Attenuation of Risks***

Upon joining the study, all staff will be required to successfully complete an NIH-approved training for research with human subjects. This training has to be completed every year by all site-based or field data collection project staff. South African-based good clinic practice (GCP) training will also need to have been up to date for all project staff. Every project staff member will complete this training immediately upon hiring and before she or he has any contact with study participants. All staff will maintain up-to-date human subjects training. Furthermore, all staff with access to project data or working with participants must sign a **Staff Agreement of Confidentiality (Exhibit 22)** and agree to comply with the terms of the project participants’ informed consent.

The following protections, against the five potential risks that were described previously, will be implemented.

1. Protections Against Possible Loss of Privacy and Disclosure of Confidential Information:

There is a possible risk for loss of privacy or disclosure of confidential information pertaining to study participants, such as information from biological tests or information participants may reveal during CHC+ intervention activities. It also is possible that project staff may reveal sensitive information to others accidentally or purposefully. Participants may reveal personal information during an intervention session that may later be disclosed by another member of the group. However, these risks are minimal because we will use procedures developed and used effectively in our previous studies, including a **Pledge of Confidentiality Form (Exhibit 23)** that participants are expected to sign before the beginning of each workshop, and reminding participants that information shared in the group is to remain confidential.

Participant confidentiality will be ensured at the data entry, storage, retrieval, and analysis stages. The risk for disclosure of information is minimal because data are de-identified. This risk will be further reduced through staff training and the development of specific procedures for protecting confidential information, such as ensuring that any biological testing is always conducted in a private space away from others. Data, including biological test results, will only be linked to personal identifying information through unique project identifiers (i.e., alphanumeric participant ID numbers). Forms with personal identifying information (e.g., full names and addresses) will be stored separately from other data in a double-locked file in a locked room at the field site, with access restricted to specific project personnel. Any data entered electronically will be transmitted through an RTI system that tracks data that have been successfully transmitted and automatically deletes data from the field computers once the data transmission is complete.

Communications software on field computers uses encrypted ***File Transfer Protocol.*** Electronic data will be kept on a server at RTI and backed up on a separate password-protected server. These risks will be further reduced through staff training and the development of specific procedures for protecting confidential information and reporting any incidents or serious adverse events (SAEs) that may occur. All staff members are trained on confidentiality and ethics with human subjects, and staff also will be educated on their responsibilities for securing and/or working on the study and will be required to sign a confidentiality agreement. This agreement specifies the consequences for violating the agreement, which may include fines, legal action, or termination of employment. Further, at the beginning and conclusion of intervention group activities, such as intervention sessions and community workshops, the facilitator will discuss the importance of confidentiality with all participants. Facilitators will encourage participants to avoid sharing personal information and emphasize that what is said in the group discussion stays within the group.

Maintaining confidentiality will be part of the ground rules for all intervention activities. However, participants also will be reminded of the exceptions—that if they share information indicating imminent harm or ongoing abuse or neglect of children, then the facilitators will be required to report this information to the authorities. Loss of privacy during data collection concerning sensitive matters may also be reduced by using ACASI data collection for self-reported data from each participant. There may be concern about the confidentiality and security of communication via telephone between the staff and the study participants to arrange follow-up visits. Staff will only use wording and language in messages that has been previously agreed on to communicate with the study participant (which is collected as part of the Locator).

1. Protections Against Psychological or Mental Discomfort:

Because of the sensitive nature of some of the topics being addressed during the proposed project, participants may experience psychological or mental discomfort related to particular issues or during certain study activities, such as receiving a positive HIV test or pregnancy test result, or topics discussed in assessments, or intervention or stigma reduction workshops. For example, discussing HIV status, previous sexual victimization or other violence could cause some distress. To reduce the risk of discomfort that may arise during study activities, participants will be informed that they can refuse to answer any questions they do not feel comfortable answering. Also, staff will be trained on how to identify emotional distress. HIV counselling and active referrals to substance abuse treatment, reproductive health, or antenatal treatment, or services for HIV assessment, and mental health counselling will reduce and mitigate this psychological distress. Standard operating procedures for handling distressed respondents, including making referrals and mandatory reporting procedures, will be established based on our previous experience in South Africa (see **Exhibit 24 Procedures for Handling Distressed Participants**). The proposed contact PI is clinically trained and the South African MPI is trained on how to handle distressed respondents. Consequently, they will train the field staff on the proper protocol for responding to distressed participants, including procedures to get assistance if a participant displays any disturbing behaviour and how to complete an incident report. Participants will be referred to counselling if necessary or if requested, and they will be asked to initial a referral form. Staff also will follow the standard outreach protocols that ensure their own safety in a situation where it could be compromised. Safety procedures will be listed in the Operations Field Manual. Staff will be tested on these procedures to ensure they understand them.

1. Procedures to Address Social Ramifications of Positive Test Results, Participation in the Study, or ART or PrEP Initiation:

Participants who are unaware that they are living with HIV or are pregnant may experience stigmatizing ramifications by sharing this information with their partner or family members. While the design of the intervention is intended to reduce episodes of violence, it is possible that couples will perpetrate violence toward one another or that other participants will experience violence from other sources during the study. Participants also may face stigmatizing ramifications if others in their community find out that they are participating in a health study or that they are taking ART or PrEP medications. If participants experience any adverse reactions to any aspects of the study, an **Incident Report (Exhibit 25)** will be filled out as soon as possible and the team will contact Drs. Wechsberg, Carney, Browne, and Dr. Orrell (if related to ART or PrEP, to assess clinic follow-up). Based on advice from senior project staff, the field staff will make appropriate referrals to medical, counselling, or other health services. SAEs will be reported within 48 hours of their occurrence by the contact MPI to NIH, the Ethics Committee in South Africa, and the Chair of the Data and Safety Monitoring Board (DSMB).

1. Potential for Interpersonal Violence Between Couples Based on Disclosure of Information:

The possibility of physical violence in response to self-disclosure of a positive HIV test result or other confidential information among couples or to another family member is a potentially serious risk. To reduce this risk, the South African MPI, who is trained in psychology, will counsel participants on strategies for safely disclosing positive HIV test results, as needed, and provide resources and referrals to support services related to violence and victimization.

1. Side Effects from PrEP or ART:

PrEP and ART will be dispensed by the clinic pharmacy and clinical staff according to South African national guidelines and with education and counselling on expected side effects. Participants, once initiated onto ART or PrEP, may have to attend follow-up clinic visits to assess tolerability and side effects of the medications. Dr. Orrell will be kept informed of any of these cases. Study staff will ensure that participants who report any side effects of ART or PrEP will be referred to their assigned clinic for further evaluation.

***Health and Safety: COVID-19***

Although the risk of COVID-19 is a new health risk that is evolving as information about safety and protection measures becomes available, we have used the available evidence and safety guidelines to develop strategies for reducing risk of COVID-19 transmission, as well as the expected trajectory of the epidemic in South Africa. We will follow guidelines provided by the South African government depending on the level of COVID-19 restrictions. Staff members will have access to personal protective equipment (PPE), which will include masks, gloves, and other protective gear such as face shields. Participants will also be given face masks. Project cars are fitted with screens and will be wiped down after transporting participants. Social distancing between the staff member and participant will be practiced—and group activities will be in venues that allow for this-if possible, outside in a private, protected space for infection control. All surfaces and equipment used will be disinfected before and after any study activities with participants are held. Furthermore, all staff, especially those who work directly with participants, will be expected to be vaccinated for their own safety as well as that of participants.

***Trial Stopping Rules***

This is a cluster-randomized study to test the efficacy of the CHC+ to increase both partners’ PrEP/ART initiation and adherence (at 3 and 6months), and reduce AOD use, sexual risk and GBV, and enhance positive gender norms and communication relative to HTS that may require stopping rules. The data collection period of the study is expected to begin in Year 2 of the study (see timeline) and with follow-ups continue through the first quarter of Year 5. During that time, we expect the DSMB to meet every 6 months for an estimated total of 8 occasions in which we will provide a p-value for a comparison of allocation by arm, attrition, as appropriate. We will let the DSMB decide if stopping rules will need to be developed. If the DSMB determines there are no stopping rules, then interim efficacy analyses will not be conducted. The structural aim of stigma-reduction training will not require stopping rules as there is minimal risk as trainings are expected as part of their professional development.

***Process of AEs and SAEs collection and reporting***

If participants experience any incidents, AEs, or SAEs in direct relation to any aspects of the study, the Project Manager will complete an Incident Report immediately and contact the South African MPI (Carney) or Medical Director (Orrell), who will contact the contact PI, Dr. Wechsberg, for additional support and to advise her of the occurrence of an SAE. Based on advice from the aforementioned project staff, the field staff will make appropriate referrals to medical, counselling and/or other health services. SAEs and Incident Reports will be reviewed immediately. SAEs will be reported within 24 hours of their occurrence by the PI to the NIDA PO, the SAMRC Ethics Committee in South Africa and the Chair of the DSMB as described previously.

Reporting procedures

All field staff members who have contact with participants in the study or with study data will be trained to react according to the aforementioned protocol in the case of an AE or SAE. Incident reports will be made by the staff member who had direct contact with the given participant with whom the AE or SAE occurred.

Reporting AEs and SAEs

All AEs and SAEs will first be reported to Dr. Carney in South Africa and to Drs. Wechsberg and Browne in the US. The Project Manager will identify, and report situations of potential distress or harm based on statements made by participants as well as observations made by field staff at the field site. Additionally, an incident report will be completed by staff, which will document any AE or SAE for future review. AEs will be reported to the NIDA Project Officer (PO) at least once per year and as a part of the annual progress report. This report will include a description of the event, when it occurred, the study arm of the participant, and the outcome or resolution. If there were no AEs, a statement that no AEs occurred will be included in the progress report or otherwise communicated to the PO. SAEs will be reported to the NIDA PO within 24 hours of the event by e-mail. This notification will include a brief explanation of the SAE and when it occurred. A written follow-up will be received within 72 hours of the event. The written follow-up will include information on the date of the event, what occurred, actions taken by project staff, planned follow-up (if any), the intervention group/study arm of the affected participant (if relevant), whether the event appeared to be related to the intervention, and whether the event affected future participation (e.g., will person continue in the study). In addition, AEs and SAEs will be reported to the South African Medical Research Council (SAMRC) (as the single IRB for this study), Ethics Committee and in reports to the DSMB.

Timeline for Reporting AEs & SAEs to NIDA (PO) and IRB:

In the event of an SAE, the PIs will ensure that these events are reported to the NIDA PO within 24 hours by e-mail and will submit a written report via e-mail to the PO no more than 2 working days later. AEs and SAEs will be reported to the SAMRC following their requirements. The Project Team will submit a written Event Report with sufficient information to SAMRC Ethics to judge whether the report raises new questions about risks to participants or the research design. Depending on the nature of the event, all reports of AEs and unanticipated problems are sent to the full IRB committee, IRB Director, IRB Chair, or an experienced IRB Member as soon as the information is obtained, and then reviewed. The reviewer(s) will decide if any corrective actions are needed, and the IRB office will communicate the outcome of the review to the Project Team. Finally, any serious unanticipated issues or serious adverse events (SAEs) will be reported within a reasonable amount of time. Deaths, even if unrelated to the project, will be sent to the DSMB Chair.

Reporting of IRB Actions to NIDA

Actions relating to AEs or SAEs will be reported to NIDA per the protocol above. Additional IRB actions, such as changes to the protocol, will be reported to NIDA in annual progress reports.

Periodic Review of and Reporting on DSMP Procedures

The Data and Safety Monitoring Plan will be reviewed as a part of the annual NIDA progress report as well as at each Data and Safety Monitoring Board meeting, to be held semi-annually. Prior to Data and Safety Monitoring Plan changes, approval will be obtained from the NIDA PO and for any major study design changes.

**Importance of the Knowledge to be Gained**

Reaching young couples in South Africa is essential to reaching the South Africa 95-95-95 goals. Including advances in HIV prevention and care and testing a gender-specific, tailored intervention for young couples who engage in HIV risk behaviours and have outside partners will be a significant opportunity to end the HIV epidemic in South Africa. Furthermore, the structural barrier of stigma in communities, especially toward young people, must also be addressed for these strategies to be effective. As noted previously, certain risks are associated with this type of research, including the risk associated with improper disclosure of sensitive information and the risk of mental discomfort of discussing topics such as HIV prevention and treatment. However, with the increased knowledge of the availability of effective treatment and prevention for HIV, the benefits of knowing one’s status and receiving appropriate prevention and medical care far outweigh these risks.

# REFERENCES

1. Sorsdahl, K., D.J. Stein, and B. Myers, *Negative attributions towards people with substance use disorders in South Africa: variation across substances and by gender.* BMC Psychiatry, 2012. **12**: p. 101.

2. Wechsberg, W.M., et al., *Substance use and sexual risk within the context of gender inequality in South Africa.* Substance Use & Misuse, 2008. **43**(8-9): p. 1186-1201.

3. Wechsberg, W.M., W.K. Luseno, and W.K. Lam, *Violence against substance-abusing South African sex workers: intersection with culture and HIV risk.* AIDS Care., 2005. **17 Suppl 1**: p. S55-64.

4. Dunkle, K., et al., *Gender-based violence, relationship power, and risk of HIV infection in women attending antenatal clinics in South Africa.* The Lancet, 2004. **363**(9419): p. 1415-1421.

5. Human Sciences Research Council (HSRC), *South African National HIV Prevalence, Incidence, Behaviour and Communication Survey, 2017: Key Findings Presentation*. 2018, HSRC Press: Cape Town.

6. Maughan-Brown, B., et al., *Age-disparate partnerships and HSV-2 among adolescent girls and young women in South Africa: implications for HIV infection risk.* Sexually Transmitted Infections, 2019. **95**(6): p. 443-448.

7. Mabaso, M., et al., *Determinants of HIV infection among adolescent girls and young women aged 15–24 years in South Africa: a 2012 population-based national household survey.* BMC Public Health, 2018. **18**(1): p. 183.

8. Maughan-Brown, B., M. Evans, and G. George, *Sexual Behaviour of Men and Women within Age-Disparate Partnerships in South Africa: Implications for Young Women's HIV Risk.* PLOS ONE, 2016. **11**(8): p. e0159162.

9. Harling, G., et al., *Do age-disparate relationships drive HIV incidence in young women? Evidence from a population cohort in rural KwaZulu-Natal, South Africa.* Journal of Aquired Immune Deficiency Syndromes 2014. **66**(4): p. 443.

10. Evans, M., et al., *HIV prevalence and ART use among men in partnerships with 15-29 year old women in South Africa: HIV risk implications for young women in age-disparate partnerships.* AIDS and Behavior., 2017. **21**(8): p. 2533-2542.

11. Joint United Nations Programme on HIV/AIDS, *HIV prevention among adolescent girls and young women*. 2016: Geneva, Switzerland.

12. Dellar, R.C., S. Dlamini, and Q.A. Karim, *Adolescent girls and young women: key populations for HIV epidemic control.* Journal of the International AIDS Society, 2015. **18**(2 Suppl 1): p. 19408.

13. Wechsberg, W.M., et al., *The male factor: outcomes from a cluster randomized field experiment with a couples-based HIV prevention intervention in a South African township.* Drug and Alcohol Dependence., 2016. **161**: p. 307-15.

14. El-Bassel, N., et al., *Effects of a couple-based intervention to reduce risks for HIV, HCV, and STIs among drug-involved heterosexual couples in Kazakhstan: a randomized controlled trial.* Journal of Acquired Immune Deficiency Syndromes., 2014. **67**(2): p. 196-203.

15. Remien, R.H., et al., *Couple-focused support to improve HIV medication adherence: a randomized controlled trial.* AIDS, 2005. **19**(8): p. 807-814.

16. Wall, K.M., et al., *Sustained effect of couples HIV counselling and testing on risk reduction among Zambian HIV serodiscordant couples.* Sexually Transmitted Infections, 2017. **93**(4): p. 259-266.

17. Conroy, A., et al., *"If she is drunk, I don't want her to take it": partner beliefs and influence on use of alcohol and antiretroviral therapy in South African couples.* AIDS and Behavior., 2017. **21**(7): p. 1885-1891.

18. Okafor, C.N., et al., *Understanding HIV risk behaviors among young men in South Africa: a syndemic approach.* AIDS and Behavior., 2018. **22**(12): p. 3962-3970.

19. Wechsberg, W.M., et al., *Substance use, gender inequity, violence and sexual risk among couples in Cape Town.* Culture, Health, and Sexualty., 2013. **15**(10): p. 1221-36.

20. South African Community Epidemiology Network on Drug Use (SACENDU), *Research Brief: Monitoring Alcohol, Tobacco and Other Drug Use Trends in South Africa (July 1996 – June 2018)*. 2019.

21. Joint United Nations Programme on HIV/AIDS (UNAIDS), *90-90-90: An ambitious treatment target to help end the AIDS epidemic*. 2014, Geneva, Switzerland: UNAIDS.

22. Eisinger, R.W., C.W. Dieffenbach, and A.S. Fauci, *HIV viral load and transmissibility of HIV infection: undetectable equals untransmittable.* Journal of the American Medical Association, 2019. **321**(5): p. 451-452.

23. Cohen, M.S., M. McCauley, and T.R. Gamble, *HIV treatment as prevention and HPTN 052.* Current Opinion in HIV and AIDS., 2012. **7**(2): p. 99-105.

24. South African National Aids Council (SANAC), *Let our actions count: South Africa’s National Strategic Plan for HIV, TB and STIs (2017-2022)*. 2017, SANAC: Pretoria.

25. Republic of South Africa Department of Health, *National Policy on HIV Pre-exposure Prophylaxis (PrEP) and Test and Treat (T&T)*. 2016. <http://www.sahivsoc.org/Files/PREP%20and%20TT%20Policy%20-%20Final%20Draft%20-%205%20May%202016%20(HIV%20news).pdf>.

26. Wechsberg, W.M., et al., *Couples only know to fight: alcohol, drug use & sex risks in informal taverns of Cape Town, the aftermath*, in *Presented at the Annual American Public Health Association Conference*. 2009, November: Philadelphia, PA.

27. Carney, T., et al., *Adolescent female school dropouts who use drugs and engage in risky sex: effects of a brief pilot intervention in Cape Town, South Africa.* AIDS Care., 2019. **31**(1): p. 77-84.

28. Psaros, C., et al., *An intervention to support HIV pre-exposure prophylaxis (PrEP) adherence in HIV serodiscordant couples in Uganda.* Journal of Acquired Immune Deficiency Syndromes., 2014. **66**(5): p. 522.

29. Curran, K., Baeten, J,, Coates, T., Kurth, A., Mugo, N., & Celum, C. *HIV-1 prevention for HIV-1 serodiscordant couples.* Current Opinion in HIV and AIDS, 2012. **9**(2): p. 160-70.

30. Haberer, J.E., et al., *Adherence to antiretroviral prophylaxis for HIV prevention: a substudy cohort within a clinical trial of serodiscordant couples in East Africa.* PLoS Med, 2013. **10**(9): p. e1001511.

31. Ware, N.C., et al., *What's love got to do with it? Explaining adherence to oral antiretroviralp pre-exposure prophylaxis (PrEP) for HIV serodiscordant couples.* Journal of Acquired Immune Deficiency Syndromes., 2012. **59**(5).

32. Dunkle, K.L., et al., *New heterosexually transmitted HIV infections in married or cohabiting couples in urban Zambia and Rwanda: an analysis of survey and clinical data.* The Lancet, 2008. **371**(9631): p. 2183-2191.

33. Morton, J.F., et al., *Counseling framework for HIV-serodiscordant couples on the integrated use of antiretroviral therapy and pre-exposure prophylaxis for HIV prevention.* Journal of Acquired Immune Deficiency Syndromes, 2017. **74 Suppl 1**: p. S15-s22.

34. Wechsberg, W.M., et al., *Substance use, sexual risk, and violence: HIV prevention intervention with sex workers in Pretoria.* AIDS Behavior., 2006. **10**(2): p. 131-7.

35. Wechsberg, W.M., et al., *Adapting the evidence-based Women's CoOp intervention to prevent human immunodeficiency virus infection in North Carolina and international settings.* North Carolina Medical Journal,, 2010. **71**(5): p. 477-481.

36. Myers, B., et al., *A trauma-informed substance use and sexual risk reduction intervention for young South African women: a mixed-methods feasibility study.* BMJ Open, 2019. **9**(2): p. e024776.

37. Wechsberg, W.M., et al., *The Young Women’s Health CoOp in Cape Town, South Africa: study protocol for a cluster-randomised trial for adolescent women at risk for HIV.* BMC Public Health, 2018. **18**(1): p. 859.

38. Wechsberg, W.M., et al., *Adapting an evidence-based HIV behavioral intervention for South African couples.* Substance Abuse Treatment, Prevention, and Policy., 2015. **10**: p. 6.

39. Bandura, A., *Social Foundations of Thought and Action*. 1986, New York: Prentice-Hall.

40. Wechsberg, W.M., Jewkes, R., Novak, S.P., et al., *A brief intervention for drug use, sexual risk behaviours and violence prevention with vulnerable women in South Africa: a randomised trial of the Women's Health CoOp.* BMJ Open. 2013. **3**(5).

41. Speizer, I.S., et al., *Changing sex risk behaviors, gender norms, and relationship dynamics among couples in Cape Town, South Africa: efficacy of an intervention on the dyad.* Social Science & Medicine., 2018. **209**: p. 95-103.

42. Belus, J.M., et al., *Investigating healthy intimate relationships in heterosexual South African couples: potential opportunities for HIV prevention efforts* in *Presented at the 51st Annual Convention of the Association for Behavioral and Cognitive Therapies, San Diego*. 2017.

43. Ndirangu, J., *PrEParing the health facility: engaging clinic staff in reducing stigma and discrimination in accessing PrEP*, in *Presented at the Ending Gender Inequalities: Evidence to Impact Conference*. 2018, October: Johannesburg, South Africa.

44. Bogart, L.M., et al., *Barriers to care among people living with HIV in South Africa: Contrasts between patient and healthcare provider perspectives.* AIDS Care., 2013. **25**(7): p. 843-53.

45. Wechsberg, W.M., Ndirangu, J., Browne, F.A., Bonner, C., Nyblade, L., Minnis, A., Speizer, I., *Addressing stigma and gender inequalities as antecedents to ending AIDS with adolescent women: Who has the power?* Presented at AIDSImpact, London, UK, 2019.

46. Were, D., Atkins, K., Musau, A., Plotkin, M., Curran, K., *Manifestations of stigma in the context of a national oral pre-exposure prophylaxis (PrEP) scale-up program in Kenya.* Presented at the 10th IAS Conference on HIV Science, Mexico City, Mexico, 2019.

47. Treves-Kagan, S., et al., *Why increasing availability of ART is not enough: a rapid, community-based study on how HIV-related stigma impacts engagement to care in rural South Africa.* BMC Public Health, 2016. **16**(1): p. 87.

48. Kidd, R., et al., *Understanding and challenging HIV stigma: Toolkit for action.* [www.icrw.org/wp-content/uploads/2016/10/Understanding-and-Challenging-HIV-Stigma-Toolkit-for-Action.pdf](http://www.icrw.org/wp-content/uploads/2016/10/Understanding-and-Challenging-HIV-Stigma-Toolkit-for-Action.pdf)*.* 2003.

49. Kidd, R., et al., *Reducing HIV stigma and gender-based violence: toolkit for health care providers in India.* [www.icrw.org/publications/reducing-hiv-stigma-and-gender-based-violence-toolkit-for-health-care-providers-in-india/](http://www.icrw.org/publications/reducing-hiv-stigma-and-gender-based-violence-toolkit-for-health-care-providers-in-india/). 2007.

50. Health Policy Project, *An intervention package for stigma-free health facilities and HIV services*. 2015, Health Policy Project: Washington, DC.

51. HIV/AIDS Alliance, *Integrating stigma reduction into HIV programming: lessons from the Africa regional stigma training programme*. 2011, HIV/AIDS Allaince: Brighton, UK.

52. Pulerwitz, J., et al., *Improving hospital-based quality of care by reducing HIV-related stigma: evaluation results from Vietnam.* AIDS and Behavior., 2015. **19**(2): p. 246-256.

53. Li, L., et al., *Implementing a stigma reduction intervention in healthcare settings.* Journal of the International AIDS Society, 2013. **16**(3 Suppl 2): p. 18710-18710.

54. Li, L., et al., *Reducing HIV-related stigma in health care settings: a randomized controlled trial in China.* American journal of public health, 2013. **103**(2): p. 286-292.

55. Srithanaviboonchai, K., et al., *Building the evidence base for stigma and discrimination-reduction programming in Thailand: development of tools to measure healthcare stigma and discrimination.* BMC Public Health, 2017. **17**(1): p. 245.

56. Batey, D.S., et al., *Adaptation and implementation of an intervention to reduce HIV-related stigma among healthcare workers in the United States: piloting of the FRESH workshop.* AIDS Patient Care STDS, 2016. **30**(11): p. 519-527.

57. Geibel, S., et al., *Stigma reduction training improves healthcare provider attitudes toward, and experiences of, young marginalized people in Bangladesh.* Journal of Adolescent Health, 2017. **60**(2, Supplement 2): p. S35-S44.

58. Friedland, B.A., et al., *Measuring intersecting stigma among key populations living with HIV: implementing the people living with HIV Stigma Index 2.0.* Journal of the International AIDS Society, 2018. **21 Suppl 5**: p. e25131.

59. Baeten JM, Donnell D, Ndase P, et al. Antiretroviral prophylaxis for HIV prevention in heterosexual men and women. *The New England Journal of Medicine.* 2012;367(5):399-410.

60. Cohen MS, Chen YQ, McCauley M, et al. Antiretroviral therapy for the prevention of HIV-1 transmission. *New England Journal of Medicine.* 2016;**375**(9):830-839.

61. Cohen MS, Chen YQ, McCauley M, et al. Prevention of HIV-1 infection with early antiretroviral therapy. *New England Journal of Medicine.* 2011;**365**(6):493-505.

62. Minnis AM, Doherty IA, Kline TL, et al. Relationship power, communication, and violence among couples: Results of a cluster-randomized HIV prevention study in a South African township. *International Journal of Women's Health.* 2015;**7:**517-525

63. Doherty I, Myers B, Zule W, et al. Seek, test and disclose: knowledge of HIV testing and serostatus among high-risk couples in a South African township. *Sexually Transmitted Infections.* 2016;**92**(1):5-11.

64. Stokols D. Translating social ecological theory into guidelines for community health promotion. *American Journal* *6*

65. Saleem HT, Narasimhan M, Denison JA, Kennedy CE. Achieving pregnancy safely for HIV-serodiscordant couples: a social ecological approach. *Journal of the International AIDS Society.* 2017;20(Suppl 1):18-23.

66. Wechsberg WM, Jones HE, Zule WA, et al. Methamphetamine (“tik”) use and its association with condom use among out-of-school females in Cape Town, South Africa. *The American Journal of Drug and Alcohol Abuse.* 2010;36(4):208-213.

66. Wechsberg WM, Bonner CP, Zule WA, et al. Addressing the nexus of risk: biobehavioral outcomes from a cluster randomized trial of the Women’s Health CoOp Plus in Pretoria, South Africa. *Drug and Alcohol Dependence.* 2019;195:16-26.

67. Wechsberg WM, Ndirangu JW, Speizer IS, et al. An implementation science protocol of the Women’s Health CoOp in healthcare settings in Cape Town, South Africa: a stepped-wedge design. *BMC Women's Health.* 2017;17(1):85.

68. Republic of South Africa Department of Health. Oral PrEP and Universal T&T: Training Workshop - PrEP Implementers 2019.

69. South African National Aids Council (SANAC). *Let our actions count: South Africa’s National Strategic Plan for HIV, TB and STIs (2017-2022).* Pretoria: SANAC;2017.

70. Castillo-Mancilla JR, Haberer JE. Adherence measurements in HIV: new advancements in pharmacologic methods and real-time monitoring. *Current HIV/AIDS reports.* 2018;15(1):49-59.

71. Celum C, Mgodi, N., Bekker, L.G., Hosek, S., Donnell, D., Anderson, P., Dye, B., Pathak, S., Agyei, Y., Fogel, J., Marzinke, M., Makgamathe, K., Kassim, S., Mukaka, S., Noble, H., Adeyeye, A., Delany-Moretlwe, S., & on behalf of the HPTN 082 Study Team,. PrEP use in young African women in HPTN 082: Effect of drug level feedback. *Presented at the 10th International AIDS Society Conference, Mexico City, Mexico.* 2019.

72. Saberi P, Ming K, Legnitto D, Neilands TB, Gandhi M, Johnson MO. Novel methods to estimate antiretroviral adherence: protocol for a longitudinal study. *Patient preference and adherence.* 2018;12:1033.

73. Raymond JF, Bucek A, Dolezal C, et al. Use of unannounced telephone pill counts to measure medication adherence among adolescents and young adults living with perinatal HIV infection. *Journal of Pediatric Psychology.* 2017;42(9):1006-1015.

74. Fredericksen R, Feldman BJ, Brown T, et al. Unannounced telephone-based pill counts: a valid and feasible method for monitoring adherence. *AIDS and behavior.* 2014;18(12):2265-2273.
